# A Mathematical Model for Smooth Muscle Cell Phenotype Switching In Atherosclerotic Plaque

**DOI:** 10.1007/s11538-026-01645-z

**Published:** 2026-04-11

**Authors:** Joseph P. Ndenda, Michael G. Watson, Ashish Misra, Mary R. Myerscough

**Affiliations:** 1 School of Mathematics and Statistics, University of Sydney, Sydney, NSW 2006 Australia; 2 School of Computational and Communication Science and Engineering, The Nelson Mandela African Institution of Science and Technology, Arusha, Tanzania; 3 School of Mathematics and Statistics, University of New South Wales, Sydney, NSW 2052 Australia; 4 Heart Research Institute, 7 Eliza Street, Newtown, NSW 2042 Australia

**Keywords:** Atherosclerosis, Smooth muscle cell, Phenotype switch, Ordinary differential equation model

## Abstract

Smooth muscle cells (SMCs) play a fundamental role in the development of atherosclerotic plaques. They ingest lipids in a similar way to monocyte-derived macrophages (MDMs) in the plaque. This can stimulate SMCs to undergo a phenotypic switch to a macrophage-like phenotype. We formulate an ordinary differential equation (ODE) model for the populations of SMCs, MDMs and smooth muscle cell-derived macrophages (SDMs) and the internalised lipid load in each population. We use this model to explore the effect on plaque fate of SMC phenotype switching. We find that when SMCs switch to a macrophage-like phenotype, there is an increase in the lipid quantity in the model plaque that is internalised inside cells. Additionally, removal of SMCs from the model plaque via phenotype switching reduces the number of SMCs in the fibrous cap, increases the lipid in the necrotic core, and increases plaque inflammation. These features are hallmarks of vulnerable plaques, whose rupture can cause heart attacks or strokes. When SDMs are highly proliferative or resistant to cell death, the model plaque becomes increasingly pathological. The model suggests that the switch of SMCs to a macrophage-like phenotype may drive the development of unstable and pathological plaques.

## Introduction

Atherosclerosis remains a major health problem and a leading cause of cardiovascular disease globally ((WHO) 2021). It is caused by chronic inflammation in the walls of large and medium-sized arteries. This inflammation results in the formation of fatty plaques (Gisterå and Hansson 2017; Bäck et al. 2019).

Cholesterol-carrying lipoproteins in the blood, mainly low-density lipoproteins (LDLs), enter the artery wall where the endothelium (the cell layer that lines the blood vessel) has become dysfunctional. These LDLs can undergo oxidative and other modifications that render them pro-inflammatory and immunogenic, and cause them to be retained in the vessel wall (Libby 2002; Goldberg and Khatib 2022). Modified LDLs (modLDL) activate resident immune cells in the intima (the part of the artery wall directly beneath the endothelium). These cells respond by secreting pro-inflammatory cytokines, which activate the endothelium and recruit circulating monocytes (Bäck et al. 2019; Libby 2002).

In the intima, monocytes differentiate into macrophages, which express scavenger receptors and internalise modLDL. Macrophages, in turn, secrete further pro-inflammatory cytokines such as tumor necrosis factor-alpha $(\text {TNF-}\alpha )$ and interleukin-1 (IL-1), which recruit more macrophages and other immune cells into the lesion (Hansson and Hermansson 2011; Zhou and Hansson 1999). The number of monocyte-derived macrophages (MDMs) in the plaque is determined by the relative rates of monocyte recruitment (Kim et al. 2020) , cell death (apoptosis) (Tabas 2010), proliferation (Lhoták et al. 2016), and emigration out of the plaque (Randolph 2008). In addition to modLDL consumption, macrophages can also acquire internalised lipid by consuming apoptotic cells (a process known as efferocytosis) (Bäck et al. 2019; Ford et al. 2019a). Macrophages can reduce their lipid burden by offloading lipid to high-density lipoprotein (HDL) particles (Brown and Goldstein 1983).

The medial layer of the artery wall (immediately beneath the intima) contains a population of vascular smooth muscle cells (SMCs). The accumulation of macrophages in the intimal layer triggers the migration of highly proliferative SMCs into the plaque (Misra et al. 2018). Recent studies using lineage tracing techniques have shown that SMC populations in plaques are either mono- or oligoclonal, which implies that very few SMCs migrate into the plaque from the media (Misra et al. 2018). The proliferative SMCs accumulate beneath the endothelium and form a fibrous cap that covers the lipid-filled plaque core. The number of SMCs in the cap is directly correlated with plaque stability (Allahverdian et al. 2018; Gomez and Owens 2012). A thin cap increases the risk of plaque rupture, which can lead to clinical complications such as heart attack or stroke.

Plaque SMCs possess the machinery to undertake phagocytosis (Liu et al. 2021), and SMCs in culture can rapidly efferocytose apoptotic SMCs (Bennett et al. 2016). This suggests that SMCs in plaques can acquire internalised lipid through ingestion of modLDL and apoptotic cells. In response to lipid loading, plaque SMCs may alter their phenotype to become macrophage-like cells (Allahverdian et al. 2012). In these SMC-derived macrophage-like cells (SDMs), SMC markers are suppressed and macrophage markers (including multiple pro-inflammatory genes) are activated (Shankman et al. 2015). In the absence of cellular lineage-tracing, it is therefore difficult to determine which cells that express macrophage markers are of SMC origin and which are of monocyte origin.

SDMs exhibit low expression of contractile markers and possess similar functions to MDMs, including innate immune signalling, phagocytosis, and efferocytosis (Bennett et al. 2016; Allahverdian et al. 2014). However, SDMs may be less effective than MDMs in clearing lipids and apoptotic cells from the lesion microenvironment, and they have a reduced phagocytic capacity compared to MDMs (Beyea et al. 2012; Wang et al. 2019). They are also known to have a significantly reduced capacity to export internalised lipid to HDL meaning that, relative to MDMs, they retain a larger proportion of the lipid that they ingest (Allahverdian et al. 2012). On the other hand, SDMs may proliferate much more rapidly than MDMs, to the extent that SMC-derived cells in plaques have been likened to tumour cells (Pan et al. 2024).

MDMs and SMC-derived cells can undergo programmed cell death in the plaque to become lipid-bearing apoptotic cells. If these apoptotic cells are not ingested and removed by living cells, they undergo secondary necrosis which leads to the formation of a necrotic core (Thorp and Tabas 2009). The necrotic core, a hallmark of advanced atherosclerosis, is associated with a high risk of thrombosis (blood clot formation) following plaque rupture (Bäck et al. 2019).

Mathematical modelling has increasingly been used to explore the cell and lipid dynamics of atherosclerotic plaque progression (El Khatib et al. 2009; Ougrinovskaia et al. 2010; Cohen et al. 2014; Bulelzai et al. 2014; Ford et al. 2019b; Chambers et al. 2023, 2025). Ford et al. (2019b) developed a system of partial integro-differential equations to model the internalised lipid load distributions in live and apoptotic plaque macrophages. The authors explored how the trafficking of lipid between these populations contributes to the long-term formation of a necrotic core. Chambers et al. (2024) extended this model to include macrophage proliferation by assuming that parent cell internalised lipid is distributed between daughter cells during division. This provides a means to reduce internalised lipid loads in plaque cells.

Several existing models have explicitly focussed on the role of SMCs in plaques. Watson et al. (2018) developed a one-dimensional multiphase model to investigate fibrous cap formation by plaque SMCs. Their findings provide insight into how SMC behaviour can influence fibrous cap thickness, but the model does not consider the impact of phenotype switching from SMCs to SDMs. Pan et al. (2021) present a two-dimensional, hybrid discrete-continuous model that considers phenotype switching of plaque SMCs. The model identifies that the SMC-to-SDM transition can reduce fibrous cap thickness and increase necrotic core size, but the phenotypic switching rate in this model is assumed to be constant and does not depend on SMC internalised lipid loads.

In this paper, we propose an ordinary differential equation (ODE) model for plaque cell and lipid dynamics that incorporates phenotypic switching of SMCs to SDMs in response to SMC lipid loading. The model accounts for the population sizes and internalised lipid loads of MDMs, SMCs, and SDMs, and explores the implications of SMC phenotypic switching for long-term plaque fate.

The remaining sections of this paper are structured as follows. The mathematical model formulation is presented in Section 2. Steady state analysis of a reduced model is presented in Section 3, followed by numerical results of the full model in Section 4. We conclude, in Section 5, by discussing the findings of this study.

## Model Formulation and Definitions

**Fig. 1:**
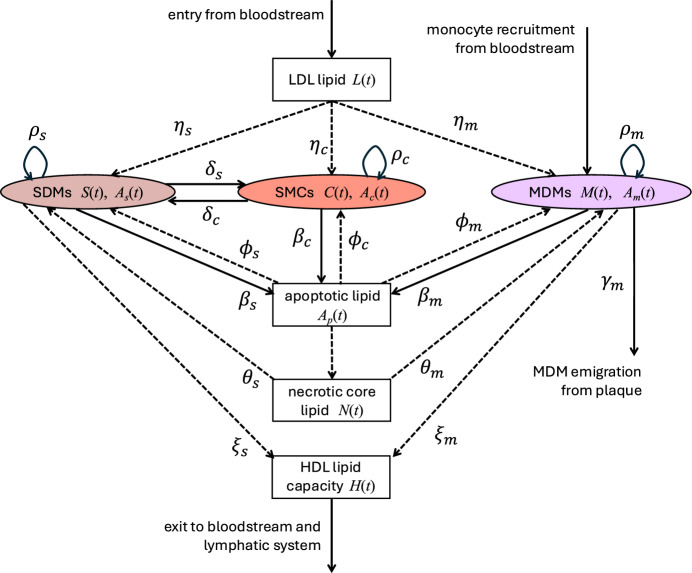
Schematic diagram showing the interactions between cell and lipid species in the model plaque. Solid arrows indicate processes that affect the dynamics of both cells and lipids; dashed arrows indicate processes that affect only the dynamics of lipids. Labels on arrows correspond to the rate constants that appear in the differential equation model

The model assumes that the plaque contains a dynamic mixture of LDL and HDL particles, MDMs, SMCs, SDMs, apoptotic cells, and necrotic core material. We let $\displaystyle {M(t)}$, $\displaystyle {C(t)}$, and $\displaystyle {S(t)}$ be time-dependent variables that represent the respective numbers of MDMs, SMCs, and SDMs. We define $\displaystyle {A_m(t)}$, $\displaystyle {A_c(t)}$, and $\displaystyle {A_s(t)}$ as the corresponding total lipid loads of these populations. The lipid inside each cell is assumed to include both the endogenous lipid $a_0$ (e.g., lipid in cell membranes), and lipid internalised from other sources. Additionally, $\displaystyle {A_p(t)}$ is the total lipid load of apoptotic cells, $\displaystyle {N(t)}$ is the lipid in the necrotic core, $\displaystyle {L(t)}$ is the total lipid on modLDL particles, and $\displaystyle {H(t)}$ is the total capacity of HDL particles to accept lipids offloaded by plaque cells. The equations that govern the evolution of these time-dependent quantities are presented below. Figure 1 presents a schematic diagram of the processes considered in the model.

### LDL and HDL

We assume that lipid on native (unmodified) LDL particles enters the artery wall at a constant rate $\Lambda \sigma _L$, where $\Lambda$ denotes the rate of serum entry into the artery wall (volume per unit time), and $\sigma _L$ denotes the lipid mass on LDL particles per unit volume of serum. Once inside the artery wall, the LDL particles are rapidly modified to become modLDL. Lipid on modLDL particles is consumed by MDMs, SDMs, and SMCs at rates $\displaystyle {\eta _m, \eta _s}$, and $\displaystyle {\eta _c}$ (per cell per unit time), respectively. With these assumptions, the dynamics of *L*(*t*) can be modelled by:

1 $$\begin{aligned} \frac{dL}{dt} = \Lambda \sigma _L -(\eta _m M+\eta _sS+\eta _cC)L. \end{aligned}$$

We assume that HDL particles also enter the artery wall at constant rate $\Lambda$, and that these particles have a fixed capacity $\displaystyle {\sigma _H}$ for lipid acceptance (lipid mass capacity per unit volume of serum). Lipid is offloaded from MDMs and SDMs to HDL particles at the respective rates $\displaystyle {\xi _m}$ and $\displaystyle {\xi _s}$ (lipid mass per cell per HDL particle per unit time). The expression of genes to promote cholesterol exporter protein ATP-binding cassette transporter A1 (ABCA1), which are needed for lipid offload to HDL, is low in SDMs compared to MDMs, so that $\displaystyle {\xi _m>\xi _s}$ (Wang et al. 2019; Cai et al. 2019). We assume that SMCs lack the machinery to offload lipid to HDL, and so $\xi _c \equiv 0$ (Allahverdian et al. 2012). The lipid mass capacity of a HDL particle when it enters the artery wall is denoted $H_0$, and we assume that HDL particles become fully loaded with lipid before leaving the artery wall. With these assumptions, the model for HDL capacity is:

2 $$\begin{aligned} \frac{dH}{dt} = \Lambda \sigma _H - (\xi _m M+\xi _sS)\frac{H}{H_0}. \end{aligned}$$

### Monocyte-Derived Macrophages (MDMs)

We assume that monocytes enter the plaque from the bloodstream in response to pro-inflammatory cytokine signals, and then rapidly differentiate into macrophages. As cytokine signals are produced in response to modLDL accumulation (Hansson 2005) and macrophage lipid loading (Allahverdian et al. 2014; Harrington 2000; Reape and Groot 1999), we assume that the rate of recruitment of MDMs into the plaque is given by:

$$\begin{aligned} f(L,M,S,A_m,A_s) = \alpha _m\,\frac{L+\tau _m(A_m - a_0 M) +\tau _s(A_s -a_0 S)}{\kappa _m + L+\tau _m(A_m - a_0 M) +\tau _s(A_s -a_0 S)}. \end{aligned}$$

Here, we assume that pro-inflammatory cytokine production in the plaque occurs proportionally to a total lipid stimulus $L+\tau _m(A_m - a_0 M) +\tau _s(A_s -a_0 S)$, and that the rate of monocyte recruitment is a saturating function of this stimulus with maximal recruitment rate $\alpha _m$. The magnitude of the lipid stimulus is a weighted sum of the total quantities of lipid in modLDL, and internalised in the MDM and SDM populations (with weightings 1, $\tau _m$, and $\tau _s$, respectively). Half-maximal recruitment occurs when the value of the lipid stimulus is equal to $\kappa _m$.

In addition to recruitment, we assume that MDMs die via apoptosis, proliferate, and emigrate out of the plaque. The dynamics of $\displaystyle {M(t)}$ are therefore modelled by:

3 $$\begin{aligned} \frac{dM}{dt} = \overbrace{f(L,M,S,A_m,A_s)}^{\text {recruitment}} + \overbrace{\rho _m M}^{\text {proliferation}} -\overbrace{\beta _m M}^{\text {apoptosis}}-\overbrace{\gamma _m M}^{\text {emigration}}, \end{aligned}$$

where $\displaystyle {\beta _m}$ is the MDM apoptosis rate, $\displaystyle {\gamma _m}$ is the MDM emigration rate, and $\displaystyle {\rho _m}$ is the MDM proliferation rate.

The dynamics of $\displaystyle {A_M(t)}$, the total lipid load of all MDMs, are modelled by:

4 $$\begin{aligned} \frac{dA_m}{dt} =&\overbrace{a_0f(L,M,S,A_m,A_s)}^{\text {recruitment}} +\overbrace{\eta _m LM}^{\text {modLDL ingestion}} - \overbrace{\frac{\xi _m HM}{H_0}}^{\text {offload to HDL}} +\overbrace{\theta _m NM}^{\text {necrotic lipid consumption}}\nonumber \\ &+\underbrace{\phi _m A_pM}_{\text {efferocytosis}} + \underbrace{a_0\rho _m M}_{\text {proliferation}}- \underbrace{\beta _mA_m}_{\text {apoptosis}}-\underbrace{\gamma A_m}_{\text {emigration}}. \end{aligned}$$

The model assumes that MDMs can internalise lipid from modLDL, apoptotic cells, and necrotic cells at rates (per cell per time) $\eta _m$, $\phi _m$, and $\theta _m$, respectively. The MDM population acquires additional lipid when new cells enter the system. This corresponds to the endogenous lipid which is either carried into the plaque by newly-recruited cells, or *de novo* synthesised during local proliferation (Chambers et al. 2024; Rodriguez Sawicki et al. 2019; Scaglia et al. 2014). The total internalised lipid in MDMs is reduced when cells emigrate, die, or efflux lipid by offloading to HDL at rate $\frac{\xi _m}{H_0}$ (per cell per time).

### Smooth Muscle Cells (SMCs) and Smooth Muscle Cell-Derived Macrophages (SDMs)

A small number of SMCs, recruited into the plaque from the media, rapidly proliferate to colonise the region beneath the endothelium (Misra et al. 2018). Exposure to lipids can stimulate these SMCs to differentiate into macrophage-like cells (Allahverdian et al. 2014), but this phenotypic change can be reversed by lipid offloading (Vengrenyuk et al. 2015). Therefore, we model the dynamics of *C*(*t*) by the equation:

5 $$\begin{aligned} \frac{dC}{dt} =&\overbrace{\rho _c \!\left( 1-\frac{C}{C_{\max }}\right) C}^{\text {proliferation}} - \overbrace{\beta _c C}^{\text {apoptosis}} - \overbrace{\delta _c C\,\frac{\left( \tfrac{A_c}{C}-a_0\right) ^n}{\alpha _c^n+\left( \tfrac{A_c}{C}-a_0\right) ^n}}^{\text {SMC-to-SDM switch}} \nonumber \\ &+ \overbrace{\delta _s S\left( 1-\frac{\left( \tfrac{A_s}{S}-a_0\right) ^n}{\alpha _s^n+\left( \tfrac{A_s}{S}-a_0\right) ^n}\right) }^{\text {SDM-to-SMC switch}}. \end{aligned}$$

The first term represents the proliferation of SMCs in the cap region, where $\displaystyle {\rho _c}$ denotes the maximum proliferation rate and $\displaystyle {C_{max}}$ denotes the carrying capacity. Physically, the value of the carrying capacity is assumed to reflect the availability of growth factors and space proximal to the endothelium (Hedin et al. 2004; Mehrhof et al. 2005). Mathematically, a growth limiting term is required to prevent unbounded SMC growth in the absence of phenotype switching. The second term in equation (5) represents SMC apoptosis at rate $\displaystyle {\beta _c}$.

We assume that the likelihood of an SMC becoming a SDM increases with lipid loading, and that the likelihood of a SDM reverting to a SMC decreases with lipid loading. This is modelled by the final two terms in (5), where $\displaystyle {\delta _c}$ is the maximum SMC-to-SDM switching rate, and $\displaystyle {\delta _s}$ is the maximum SDM-to-SMC switching rate. The overall switching rates are assumed to be functions of the ingested lipid per cell. That is, $(\frac{A_c}{C} - a_0)$ and $(\frac{A_s}{S} - a_0)$ for the forward and backward switching, respectively. We use a Hill function formulation to express the fact that the SMC-to-SDM phenotype switch is unlikely if average SMC lipid is low, and the SDM-to-SMC phenotype switch is unlikely if average SDM lipid is high. The exponent $n\geqslant 1$ controls the sharpness of the switch. The average ingested lipid loads for a half-maximal switching rate are $\alpha _c$ and $\alpha _s$, where we assume $\alpha _c>\alpha _s$.

Plaque SMCs express various receptors that mediate modLDL uptake (Liu et al. 2021). SMCs in culture also rapidly ingest apoptotic SMCs (Bennett et al. 2016). We therefore assume that the total lipid load of the SMC population $\displaystyle {A_c(t)}$ has dynamics given by:

6 $$\begin{aligned} \frac{dA_c}{dt} =&\overbrace{\eta _c L C}^{\text {modLDL ingestion}} + \overbrace{\phi _c A_p C}^{\text {efferocytosis}} + \overbrace{a_0 \rho _c \!\left( 1-\frac{C}{C_{\max }}\right) C}^{\text {proliferation}} - \overbrace{\beta _c A_c}^{\text {apoptosis}} \nonumber \\&\quad - \underbrace{\delta _c A_c\,\frac{\left( \tfrac{A_c}{C}-a_0\right) ^n}{\alpha _c^n+\left( \tfrac{A_c}{C}-a_0\right) ^n}}_{\text {SMC-to-SDM switch}} + \underbrace{\delta _s A_s\left( 1-\frac{\left( \tfrac{A_s}{S}-a_0\right) ^n}{\alpha _s^n+\left( \tfrac{A_s}{S}-a_0\right) ^n}\right) }_{\text {SDM-to-SMC switch}}, \end{aligned}$$

where $\eta _c$ and $\phi _c$ are the rates (per cell per time) of lipid ingestion from modLDL and apoptotic cells, respectively. As for MDMs, we assume that proliferating SMCs *de novo* generate endogenous lipid for their daughter cells. This produces the proliferation term in equation (6). The remaining terms in equation (6) represent the lipid lost to the apoptotic lipid pool upon SMC apoptosis, the lipid transferred to the SDM population upon SMC-to-SDM phenotypic switching, and the lipid regained from the SDM population upon SDM-to-SMC phenotypic switching. We remark that, for equation (6) to remain physically valid, we must avoid the ambiguity of “negative proliferation” that arises if $C(t)>C_{max}$. For the model as a whole, we therefore impose the notional restriction that $C(t)\leqslant C_{max}$ for all $t\geqslant 0$.

The SDM population dynamics share features of the MDM and SMC populations. The SDMs are subject to phenotypic switching, proliferation (at rate $\rho _s$), and apoptosis (at rate $\beta _s$), and we assume that they do not emigrate (Liu et al. 2021; Bennett et al. 2016). The SDM dynamics *S*(*t*) are modelled by the equation:

7 $$\begin{aligned} \frac{dS}{dt} = \overbrace{\delta _c C\,\frac{\left( \tfrac{A_c}{C}-a_0\right) ^n}{\alpha _c^n+\left( \tfrac{A_c}{C}-a_0\right) ^n}}^{\text {SMC-to-SDM switch}} + \overbrace{\rho _s S}_{\text {proliferation}} - \overbrace{\beta _s S}^{\text {apoptosis}} - \overbrace{\delta _s S\left( 1-\frac{\left( \tfrac{A_s}{S}-a_0\right) ^n}{\alpha _s^n+\left( \tfrac{A_s}{S}-a_0\right) ^n}\right) }^{\text {SDM-to-SMC switch}}. \end{aligned}$$

We assume that SDMs ingest lipid from modLDL, apoptotic cells, and necrotic material at rates (per cell per time) $\eta _s$, $\phi _s$, and $\theta _s$, respectively. We also assume that SDMs can offload ingested lipid to HDL at rate $\frac{\xi _s}{H_0}$ (per cell per time). The dynamics of the total lipid load of the SDM population $A_s(t)$ therefore follow:

8 $$\begin{aligned} \frac{dA_s}{dt} =&\overbrace{\eta _s L S}^{\text {modLDL ingestion}} - \overbrace{\tfrac{\xi _s H S}{H_0}}^{\text {HDL offloading}} + \overbrace{\theta _s N S}^{\text {necrotic lipid consumption}} + \overbrace{\phi _s A_p S}^{\text {efferocytosis}} \nonumber \\ &+ \underbrace{\delta _c A_c\,\frac{\left( \tfrac{A_c}{C}-a_0\right) ^n}{\alpha _c^n+\left( \tfrac{A_c}{C}-a_0\right) ^n}}_{\text {SMC-to-SDM switch}} + \underbrace{a_0 \rho _s S}_{\text {proliferation}} - \underbrace{\beta _s A_s}_{\text {apoptosis}} - \underbrace{\delta _s A_s\left( 1-\frac{\left( \tfrac{A_s}{S}-a_0\right) ^n}{\alpha _s^n+\left( \tfrac{A_s}{S}-a_0\right) ^n}\right) }_{\text {SDM-to-SMC switch}}. \end{aligned}$$

In the above equation, we assume that the parameters $\eta _s$, $\phi _s$ and $\theta _s$ all have smaller values than the corresponding parameters ($\eta _m$, $\phi _m$, $\theta _m$) for MDMs.

### Apoptotic Lipid and Necrotic Core Lipid

We assume that all cell types contribute to a single class of apoptotic cells when they die. As such, a cell does not “know” whether the apoptotic lipid it ingests was originally contained in a MDM, SMC, or SDM. All apoptotic cells undergo secondary necrosis at rate $\displaystyle {\nu }$ if not ingested by another live cell. The total mass of lipid in the apoptotic lipid pool $A_p(t)$ is therefore governed by the equation:

9 $$\begin{aligned} \frac{dA_p}{dt} = \beta _m A_m + \beta _s A_s + \beta _c A_c - \left( \phi _m M + \phi _s S + \phi _c C\right) A_p - \nu A_p, \end{aligned}$$

where the first three terms model the accumulation of apoptotic lipid due to plaque cell apoptosis, the following three terms model plaque cell ingestion of apoptotic lipid, and the final term models the loss of apoptotic lipid due to secondary necrosis of apoptotic cells.

The corresponding dynamics of the necrotic lipid mass *N*(*t*) are given by the equation:

10 $$\begin{aligned} \frac{dN}{dt} = \nu A_p - \left( \theta _m M + \theta _s S\right) N, \end{aligned}$$

where the first term represents necrotic lipid generation due to secondary necrosis of apoptotic cells, and the final two terms represent necrotic lipid consumption by MDMs and SDMs. It is assumed that SMCs do not consume necrotic lipid as plaque cell necrosis occurs mostly in the core of the plaque, which is distal to the cap region where SMCs accumulate.

### Initial Conditions and Model Parameterisation

At time $t=0$, the equations (1)–(10) are subject to the initial conditions:

11 $$\begin{aligned} L(0)=H(0)=M(0)=A_m(0)=C(0)=A_c(0)=S(0)=A_s(0)=A_p(0)=N(0)=0. \end{aligned}$$

From these zero initial conditions, plaque formation is initiated by an influx of LDL lipid which, in turn, stimulates the recruitment of MDMs. No SMCs, nor SDMs, enter the plaque at this stage. Consistent with experimental observations, we assume that SMCs first enter the plaque several weeks after the MDMs (Misra et al. 2018). Thus, at time $t=t_{c}>0$, we introduce a small population of SMCs containing only their endogeneous lipid by setting:

12 $$\begin{aligned} C(t_c)=C_{init}, \,\text { and }\, A_c(t_c)=a_0\,C_{init}. \end{aligned}$$

The subsequent growth of this SMC population by proliferation provides a potential source of SDMs via phenotypic switching.

A comprehensive effort has been made supply the model with accurate parameter estimates based on available experimental data. See Table 1 for a summary of the dimensional parameters used in the model and references to studies from which estimates were obtained.

**Table 1:** Summary of dimensional model parameters

Parameter	Description	Value	Unit	Source
$\alpha _m$	MDMs maximum recruitment rate	5.4	cell/hour	(Swirski et al. 2006)
$\kappa _m$	Lipid stimulus for half-maximal MDM recruitment	$5\times 10^{-8}$	g	
$\rho _m$	MDMs proliferation rate	0.0005	per hour	(Robbins et al. 2013; Tang et al. 2015)
$\beta _m$	MDMs apoptosis rate	0.002	per hour	(Yona et al. 2013)
$\gamma$	MDMs emigration rate	0.0015	per hour	(Williams et al. 2018; Lee et al. 2019)
$a_0$	Mass of endogenous lipid in each cell	$26.6\times 10^{-12}$	g/cell	(Sokol et al. 1991; Cooper and Adams 2022)
$\rho _c$	SMCs proliferation rate	0.016	per hour	(Misra et al. 2018)
$C_{max}$	SMCs maximum carrying capacity	750	cells	
$\beta _c$	SMCs apoptosis rate	0.0004	per hour	
$\delta _c$	SMCs maximum switching rate to SDMs	0.006	per hour	
$\alpha _c$	SMC ingested lipid load for half-maximal switching to SDM	$2a_0$	g/cell	
$\rho _s$	SDMs proliferation rate	$0.625\rho _c$	per hour	(Misra et al. 2018)
$\beta _s$	SDMs apoptosis rate	$1.06\rho _s$	per hour	(Shankman et al. 2015)
$\delta _s$	SDMs maximum switching rate to SMCs	$\delta _c$	per hour	
$\alpha _s$	SDM ingested lipid load for half-maximal switching to SMC	$0.5a_0$	g/cell	
$\Lambda$	Rate of serum entry into artery wall	$5\times 10^{-4}$	$\mu$L/hour	(Nielsen 1996)
$\sigma _L$	Lipid content of LDL particles per unit volume of serum	$8\times 10^{-7}$	g/$\mu$L	(Lee et al. 2012; Orlova et al. 1999)
$\sigma _H$	Lipid capacity of HDL particles per unit volume of serum	$5\times 10^{-7}$	g/$\mu$L	(Casula et al. 2021)
$H_0$	Maximum lipid capacity of single HDL particle	$5\times 10^{-17}$	g/HDL particle	(Kontush et al. 2015; Matyus et al. 2015)
$\eta _m$	modLDL consumption rate by MDMs	$1\times 10^{-6}$	per cell per hour	
$\eta _c$	modLDL consumption rate by SMCs	$0.4\eta _m$	per cell per hour	
$\eta _s$	modLDL consumption rate by SDMs	$0.75\eta _m$	per cell per hour	(Vengrenyuk et al. 2015)
$\xi _m$	MDM offloading rate of lipid to HDL	$1\times 10^{-23}$	g/cell per HDL particle per hour	
$\xi _s$	SDM offloading rate of lipid to HDL	$0.25\xi _m$	g/cell per HDL particle per hour	(Vengrenyuk et al. 2015)
$\phi _m$	Apoptotic lipid consumption rate by MDMs	$1\times 10^{-5}$	per cell per hour	(Ford et al. 2019b, a)
$\phi _c$	Apoptotic lipid consumption rate by SMCs	$0.2\phi _m$	per cell per hour	
$\phi _s$	Apoptotic lipid consumption rate by SDMs	$0.25\phi _m$	per cell per hour	(Vengrenyuk et al. 2015)
$\theta _m$	Necrotic lipid consumption rate by MDMs	$3.6\times 10^{-6}$	per cell per hour	(Schrijvers et al. 2005)
$\theta _s$	Necrotic lipid consumption rate by SDMs	$0.25\theta _m$	per cell per hour	(Vengrenyuk et al. 2015)
$\nu$	Secondary necrosis rate	0.05	per hour	(Ford et al. 2019b; Saraste and Pulkki 2000)
$t_c$	Time of SMC entry into plaque	850	hours	(Misra et al. 2018)
$C_{init}$	SMC population size at time $t=t_c$	1.35	cells	(Misra et al. 2018)

### Measures of Model Plaque Pathology

The model plaques that are generated by solving the above equations can all be considered pathological to some degree – they represent mature plaques that have progressed to the stage of SMC infiltration. However, the extent of this pathology will naturally vary as parameter values are modified. As is the case for real atherosclerotic plaques, the current model admits no single metric that can uniquely describe the extent of model plaque pathology. Instead, we consider that the key features of a highly pathological model plaque are: (1) a large macrophage population, $M(t)+S(t)$; (2) a small cap SMC population, *C*(*t*); and (3) a large necrotic lipid mass, *N*(*t*). These factors can be linked, respectively, to (1) high-grade inflammation; (2) a thin fibrous cap; and (3) a large necrotic core, which are the features most frequently used to classify instability and vulnerability to rupture in real atherosclerotic plaques (Virmani et al. 2000).

### Nondimensionalisation

We rewrite the model equations (1)–(10) in terms of the following dimensionless variables, denoted with tildes:

$$\begin{aligned} \tilde{t}:= \beta _m t,\quad \tilde{C}:=\frac{\beta _m}{\alpha _m}C,\quad \tilde{S}:=\frac{\beta _m}{\alpha _m}S,\quad \tilde{M}:=\frac{\beta _m}{\alpha _m}M,\quad \tilde{L}:=\frac{\beta _m}{a_0\alpha _m}L,\quad \tilde{H}:=\frac{\beta _m}{a_0\alpha _m}H, \nonumber \\ \tilde{A_c}:=\frac{\beta _m}{a_0\alpha _m}A_c, \quad \tilde{A_s}:=\frac{\beta _m}{a_0\alpha _m}A_s\quad \tilde{A}_m:=\frac{\beta _m}{a_0\alpha _m}A_m, \quad \tilde{A_p}:=\frac{\beta _m}{a_0\alpha _m}A_p, \quad \tilde{N}:=\frac{\beta _m}{a_0\alpha _m}N. \end{aligned}$$

The corresponding dimensionless model parameters are defined in Table 2.

Dropping the tildes for notational convenience, we have the following non-dimensional ODE system:

13a $$\begin{aligned} \frac{dL}{dt}&=\sigma _L -(\eta _mM+\eta _sS+\eta _cC)L\end{aligned}$$

13b $$\begin{aligned} \frac{dH}{dt}&=\sigma _H -(\zeta _mM+\zeta _sS)H\end{aligned}$$

13c $$\begin{aligned} \frac{dC}{dt}&= \rho _c\left( 1-\frac{C}{C_0}\right) C - \beta _c C - \frac{\delta _c C\left( \tfrac{A_c}{C}-1\right) ^n}{\alpha _c^n+\left( \tfrac{A_c}{C}-1\right) ^n} + \frac{\delta _s\alpha _s^n S}{\alpha _s^n+\left( \tfrac{A_s}{S}-1\right) ^n} \end{aligned}$$

13d $$\begin{aligned} \frac{dA_c}{dt}&= \eta _c LC+\Phi _c A_pC+ \rho _c\left( 1-\frac{C}{C_0}\right) C - \beta _c A_c -\frac{\delta _c A_c\left( \tfrac{A_c}{C}-1\right) ^n}{\alpha _c^n+\left( \tfrac{A_c}{C}-1\right) ^n} + \frac{\delta _s\alpha _s^n A_s}{\alpha _s^n+\left( \tfrac{A_s}{S}-1\right) ^n}\end{aligned}$$

13e $$\begin{aligned} \frac{dS}{dt}&= \frac{\delta _c C\left( \tfrac{A_c}{C}-1\right) ^n}{\alpha _c^n+\left( \tfrac{A_c}{C}-1\right) ^n} + \rho _s S - \beta _s S - \frac{\delta _s\alpha _s^n S}{\alpha _s^n+\left( \tfrac{A_s}{S}-1\right) ^n}\end{aligned}$$

13f $$\begin{aligned} \frac{dA_s}{dt}&= \left( \eta _sL-\zeta _sH+\Phi _s A_p +\Theta _s N + \rho _s \right) S - \beta _s A_s + \frac{\delta _c A_c\left( \tfrac{A_c}{C}-1\right) ^n}{\alpha _c^n+\left( \tfrac{A_c}{C}-1\right) ^n} - \frac{\delta _s\alpha _s^n A_s}{\alpha _s^n+\left( \tfrac{A_s}{S}-1\right) ^n}\end{aligned}$$

13g $$\begin{aligned} \frac{dM}{dt}&= \frac{L+\tau _m(A_m-M)+\tau _s(A_s-S)}{\Gamma +L+\tau _m(A_m-M)+\tau _s(A_s-S)} + \rho _m M - M - \gamma M\end{aligned}$$

13h $$\begin{aligned} \frac{dA_m}{dt}&= \frac{L+\tau _m(A_m-M)+\tau _s(A_s-S)}{\Gamma +L+\tau _m(A_m-M)+\tau _s(A_s-S)}\nonumber \\&\quad +\left( \eta _mL-\zeta _mH+\Phi _m A_p +\Theta _m N + \rho _m \right) M - \left( 1+ \gamma \right) A_m\end{aligned}$$

13i $$\begin{aligned} \frac{dA_p}{dt}&= A_m + \beta _s A_s + \beta _c A_c - \left( \Phi _m M + \Phi _s S + \Phi _c C\right) A_p - \nu A_p\end{aligned}$$

13j $$\begin{aligned} \frac{dN}{dt}&= \nu A_p - \left( \Theta _m M + \Theta _s S\right) N \end{aligned}$$

These equations are once again subject to zero initial conditions at time $t=0$. SMCs are introduced into the system at time $t=t_c>0$ according to the following conditions:

14 $$\begin{aligned} C(t_c)=A_c(t_c)=C_{init}. \end{aligned}$$

We remark that the proof of existence of solutions for the system (13) remains an open problem. However, given that the ODEs primarily contain linear or bilinear terms, with a few bounded and continuous nonlinear terms, we anticipate the existence of physically meaningful solutions for all time with the initial conditions that we impose. In all that follows, we therefore assume that solutions do indeed exist.

**Table 2:** Summary of dimensionless model parameters

Parameter	Definition	Description	Approx. Value
$\displaystyle {\tilde{\rho }_c}$	$\displaystyle {\frac{\rho _c}{\beta _m}}$	SMCs proliferation rate	8
$\displaystyle {\tilde{C_0}}$	$\displaystyle {\frac{\beta _mC_{max}}{\alpha _m}}$	SMCs maximum carrying capacity	0.28
$\displaystyle {\tilde{\beta }_c}$	$\displaystyle {\frac{\beta _c}{\beta _m}}$	SMCs apoptosis rate	0.2
$\displaystyle {\tilde{\delta }_c}$	$\displaystyle {\frac{\delta _c}{\beta _m}}$	Maximum SMC-to-SDM phenotype switching rate	3
$\displaystyle {\tilde{\alpha }_c}$	$\displaystyle {\frac{\alpha _c}{a_0}}$	Lipid load for half-maximal SMC-to-SDM switching	2
$\displaystyle {\tilde{\delta }_s}$	$\displaystyle {\frac{\delta _s}{\beta _m}}$	Maximum SDM-to-SMC phenotype switching rate	3
$\displaystyle {\tilde{\alpha }_s}$	$\displaystyle {\frac{\alpha _s}{a_0}}$	Lipid load for half-maximal SDM-to-SMC switching	0.5
$\displaystyle {\tilde{\sigma }_L}$	$\displaystyle {\frac{\Lambda \sigma _L}{a_0\alpha _m}}$	Net influx rate of lipids on LDL	2.78
$\displaystyle {\tilde{\sigma }_H}$	$\displaystyle {\frac{\Lambda \sigma _H }{a_0\alpha _m}}$	Net influx rate of HDL lipid efflux capacity	1.74
$\displaystyle {\Phi _m}$	$\displaystyle {\frac{\phi _m\alpha _m}{\beta _m^2}}$	MDM apoptotic lipid consumption rate	13.5
$\displaystyle {\Phi _s}$	$\displaystyle {\frac{\phi _s\alpha _m}{\beta _m^2}}$	SDM apoptotic lipid consumption rate	3.38
$\displaystyle {\Phi _c}$	$\displaystyle {\frac{\phi _c\alpha _m}{\beta _m^2}}$	SMC apoptotic lipid consumption rate	2.7
$\displaystyle {\Theta _m}$	$\displaystyle {\frac{\theta _m\alpha _m}{\beta _m^2}}$	MDM necrotic lipid consumption rate	4.86
$\displaystyle {\Theta _s}$	$\displaystyle {\frac{\theta _s\alpha _m}{\beta _m^2}}$	SDM necrotic lipid consumption rate	1.22
$\displaystyle {\tilde{\eta }_m}$	$\displaystyle {\frac{\alpha _m\eta _m}{\beta _m^2}}$	MDM modLDL consumption rate	1.35
$\displaystyle {\tilde{\eta }_s}$	$\displaystyle {\frac{\alpha _m\eta _s}{\beta _m^2}}$	SMC modLDL consumption rate	1.01
$\displaystyle {\tilde{\eta }_c}$	$\displaystyle {\frac{\alpha _m\eta _c}{\beta _m^2}}$	SDM modLDL consumption rate	0.54
$\displaystyle {\zeta _m}$	$\displaystyle {\frac{\alpha _m\xi _m}{H_0\beta _m^2}}$	MDM offloading rate to HDL	0.27
$\displaystyle {\zeta _s}$	$\displaystyle {\frac{\alpha _m\xi _s}{H_0\beta _m^2}}$	SDM offloading rate to HDL	0.07
$\displaystyle {\tilde{\nu }}$	$\displaystyle {\frac{\nu }{\beta _m}}$	Secondary necrosis rate	25
$\displaystyle {\tilde{\rho }_s}$	$\displaystyle {\frac{\rho _s}{\beta _m}}$	SDM proliferation rate	5
$\displaystyle {\tilde{\beta }_s}$	$\displaystyle {\frac{\beta _s}{\beta _m}}$	SDM apoptosis rate	5.3
$\displaystyle {\tilde{\Gamma }}$	$\displaystyle {\frac{\kappa _m\beta _m}{a_0\alpha _m}}$	Lipid stimulus for half-maximal MDM recruitment	0.70
$\displaystyle {\tilde{\rho }_m}$	$\displaystyle {\frac{\rho _m}{\beta _m}}$	MDM proliferation rate	0.25
$\displaystyle {\tilde{\gamma }}$	$\displaystyle {\frac{\gamma }{\beta _m}}$	MDM emigration rate	0.75
$\displaystyle {\tilde{t}_c}$	$\displaystyle {\beta _m t_c}$	Time of SMC entry into plaque	1.7
$\displaystyle {\tilde{C}_{init}}$	$\displaystyle {\frac{\beta _mC_{init}}{\alpha _m}}$	SMC population at time $\tilde{t}=\tilde{t}_c$	$5\times 10^{-4}$
$\tau _m$	-	MDM lipid stimulus weighting for MDM recruitment	1
$\tau _s$	-	SDM lipid stimulus weighting for MDM recruitment	1
*n*	-	Hill coefficient in switching functions	4

## Reduction to Three Equations—Dynamics of SMC Phenotypic Switching

In this section, we use a reduced version of the system (13) to study SMC phenotypic switching in isolation from the other processes in the model plaque. To make this feasible, we set $\delta _s = 0$ and assume that SMC lipid uptake occurs at the constant rate $\Pi _c>0$, which is independent of *L*(*t*) and $A_p(t)$. This decouples equations (13c) to (13e) from the rest of the system and allows us to analyse the long-term dynamics of the SMC and SDM populations in the presence of SMC lipid loading. The dimensionless equations of interest in this case are:

15a $$\begin{aligned} \frac{dC}{dt}&= \rho _c C\left( 1-\frac{C}{C_0}\right) - \beta _c C - \delta _c C \frac{ \left( \frac{A_c}{C}-1\right) ^n}{\alpha _c^n+\left( \frac{A_c}{C}-1\right) ^n}, \end{aligned}$$

15b $$\begin{aligned} \frac{dA_c}{dt}&= \Pi _cC + \rho _c C\left( 1-\frac{C}{C_0}\right) - \beta _c A_c - \delta _c A_c\frac{ \left( \frac{A_c}{C}-1\right) ^n}{\alpha _c^n+\left( \frac{A_c}{C}-1\right) ^n}, \end{aligned}$$

15c $$\begin{aligned} \frac{dS}{dt}&= \delta _c C \frac{ \left( \frac{A_c}{C}-1\right) ^n}{\alpha _c^n+\left( \frac{A_c}{C}-1\right) ^n} - \left( \beta _S - \rho _S\right) S. \end{aligned}$$

We note that equation (15c) for the SDM population only supports a non-trivial steady state solution if $\beta _S-\rho _S>0$. Otherwise, any supply of SDMs from phenotypic switching of SMCs leads to unbounded growth of *S*(*t*). In what follows, we therefore assume $\beta _S>\rho _S$.

Steady state analysis of the system (15) can be simplified by replacing the equation for the total lipid load of all SMCs, $A_c$, with the corresponding equation for the average SMC lipid load, $\bar{A}_c = \frac{A_c}{C}\geqslant 1$. We therefore study the alternative system:

16a $$\begin{aligned} \frac{dC}{dt}&= \rho _cC\left( 1-\frac{C}{C_0}\right) - \beta _c C - \delta _cC\frac{ \left( \bar{A}_c-1\right) ^n}{\alpha _c^n+\left( \bar{A}_c-1\right) ^n}, \end{aligned}$$

16b $$\begin{aligned} \frac{d\bar{A}_c}{dt}&= \Pi _c - \rho _c\left( 1-\frac{C}{C_0}\right) \left( \bar{A}_c-1\right) , \end{aligned}$$

16c $$\begin{aligned} \frac{dS}{dt}&= \delta _cC\frac{ \left( \bar{A}_c-1\right) ^n}{\alpha _c^n+\left( \bar{A}_c-1\right) ^n} - \left( \beta _S - \rho _S\right) S. \end{aligned}$$

The system (16) has two steady state solutions $\left( C^*,\,\bar{A}_c^*,\,S^*\right)$. The first is:

17 $$\begin{aligned} \Bigg (C^*_1 = 0, \;\; \bar{A}_{c,1}^* = 1+\frac{\Pi _c}{\rho _c}, \;\; S^*_1 = 0\Bigg ). \end{aligned}$$

This corresponds to the trivial steady state $\left( C^*=0,\,A_c^*=0,\,S^*=0\right)$ of the system (15), and indicates that, on approach to this steady state, the average ingested lipid load maintains a non-zero value. Assuming that $C^*\ne 0$, the second steady state solution of (16) is:

18 $$\begin{aligned} \Bigg (\,C^*_2 = C_0 \left[ \,1-\frac{\Pi _c}{k\rho _c}\,\right] , \;\; \bar{A}_{c,2}^* = 1+k, \;\; S^*_2 = \frac{C^*_2}{\beta _s - \rho _s} \left[ \,\frac{\Pi _c}{k} - \beta _c\,\right] \,\Bigg ), \end{aligned}$$

where $k>0$ denotes the average ingested SMC lipid load at steady state, and corresponds to the real, positive solution of the equation:

19 $$\begin{aligned} \left( \beta _c+\delta _c\right) k^{n+1}-\Pi _ck^n+\alpha _c^n\beta _ck-\alpha _c^n\Pi _c = 0. \end{aligned}$$

The steady state solution (18) is positive and, hence, physical provided that:

20 $$\begin{aligned} \rho _c>\frac{\Pi _c}{k}, \end{aligned}$$

where:

21 $$\begin{aligned} \frac{\Pi _c}{k}=\beta _c+\delta _c\,\frac{k^n}{\alpha _c^n+k^n}, \end{aligned}$$

such that $\frac{\Pi _c}{k}\in \left( \beta _c,\,\beta _c+\delta _c\right)$. We note that, in the special case $n=1$, equation (19) is a quadratic in *k* that has the positive solution:

22 $$\begin{aligned} k = \frac{\Pi _c-\beta _c\alpha _c+\sqrt{\left( \Pi _c+\beta _c\alpha _c\right) ^2+4\delta _c\Pi _c\alpha _c}}{2\left( \beta _c+\delta _c\right) }. \end{aligned}$$

To determine the stability of the steady state solutions (17) and (18), we derive the Jacobian matrix $J\left( C,\bar{A}_c,S\right)$ of the system (16). The Jacobian matrix is:

23 $$\begin{aligned} J\left( C,\bar{A}_c,S\right) = \begin{bmatrix} \;\rho _c\left( 1-\frac{2C}{C_0}\right) - \beta _c -\delta _c\frac{ \left( \bar{A}_c-1\right) ^n}{\alpha _c^n+\left( \bar{A}_c-1\right) ^n} & \;\;-\frac{n\alpha _c^n \delta _cC\left( \bar{A}_c-1\right) ^{n-1}}{\left( \alpha _c^n+\left( \bar{A}_c-1\right) ^n\right) ^2}\;\; & 0 \\ \frac{\rho _c}{C_0}\left( \bar{A}_c-1\right) & \rho _c\left( \frac{C}{C_0}-1\right) & 0 \\ \delta _c\frac{ \left( \bar{A}_c-1\right) ^n}{\alpha _c^n+\left( \bar{A}_c-1\right) ^n} & \frac{n\alpha _c^n \delta _cC\left( \bar{A}_c-1\right) ^{n-1}}{\left( \alpha _c^n+\left( \bar{A}_c-1\right) ^n\right) ^2} & \rho _s-\beta _s\; \end{bmatrix}. \end{aligned}$$

Evaluating the Jacobian at the non-trivial steady state (18) gives:

24 $$\begin{aligned} J\left( C^*_2,\bar{A}_{c,2}^*,S^*_2\right) = \begin{bmatrix} \;\frac{\Pi _c}{k}-\rho _c & \;\;-\frac{n\alpha _c^n \delta _ck^{n-1}C_0\left( 1-\frac{\Pi _c}{k\rho _c}\right) }{\left( \alpha _c^n+k^n\right) ^2}\;\; & 0 \\ \frac{k\rho _c}{C_0} & -\frac{\Pi _c}{k} & 0 \\ \frac{\Pi _c}{k}-\beta _c & \frac{n\alpha _c^n \delta _ck^{n-1}C_0\left( 1-\frac{\Pi _c}{k\rho _c}\right) }{\left( \alpha _c^n+k^n\right) ^2} & \rho _s-\beta _s\; \end{bmatrix}. \end{aligned}$$

This matrix has the eigenvalue $\lambda =\rho _s-\beta _s<0$. The remaining eigenvalues are determined by the entries in the upper left $2\times 2$ matrix, which we denote $J_2\left( C^*_2,\bar{A}_{c,2}^*\right)$. Assuming that $\rho _c>\frac{\Pi _c}{k}$, we have $\text {tr}J_2\left( C^*_2,\bar{A}_{c,2}^*\right) <0$, and $\text {det}J_2\left( C^*_2,\bar{A}_{c,2}^*\right) >0$, which implies that both eigenvalues of the matrix $J_2\left( C^*_2,\bar{A}_{c,2}^*\right)$ have negative real parts. Hence, the steady state solution (18) is stable when it is physical because all three eigenvalues of $J\left( C^*_2,\bar{A}_{c,2}^*,S^*_2\right)$ have negative real parts.

Evaluating the Jacobian at the trivial steady state (17) gives:

25 $$\begin{aligned} J\left( C^*_1,\bar{A}_{c,1}^*,S^*_1\right) = \begin{bmatrix} \;\rho _c - \beta _c -\delta _c\frac{\left( \frac{\Pi _c}{\rho _c}\right) ^n}{\alpha _c^n+\left( \frac{\Pi _c}{\rho _c}\right) ^n} & 0 & 0 \\ \frac{\Pi _c}{C_0} & \;\;-\rho _c\;\; & 0 \\ \delta _c\frac{\left( \frac{\Pi _c}{\rho _c}\right) ^n}{\alpha _c^n+\left( \frac{\Pi _c}{\rho _c}\right) ^n} & 0 & \rho _s-\beta _s\; \end{bmatrix}. \end{aligned}$$

This matrix has the real eigenvalues $\lambda _1=\rho _c - \beta _c -\delta _c\frac{\left( \frac{\Pi _c}{\rho _c}\right) ^n}{\alpha _c^n+\left( \frac{\Pi _c}{\rho _c}\right) ^n}$, $\lambda _2=-\rho _c$, and $\lambda _3 = \rho _s - \beta _s$. The steady state solution (17) is therefore an unstable saddle point when $\lambda _1>0$, and a stable node when $\lambda _1<0$. Using the condition (20) and equation (21), we deduce that $\lambda _1>0$ when the steady state (18) is physical, and $\lambda _1<0$ otherwise. This analysis implies that the model SMC population will die out in the long-term if the rate of SMC proliferation is insufficient to dominate the combined rates of apoptosis and phenotype switching. The absence of SMCs removes the supply of new SDMs, so the model SDM population will also die out in the long-term in this case.

**Fig. 2:**
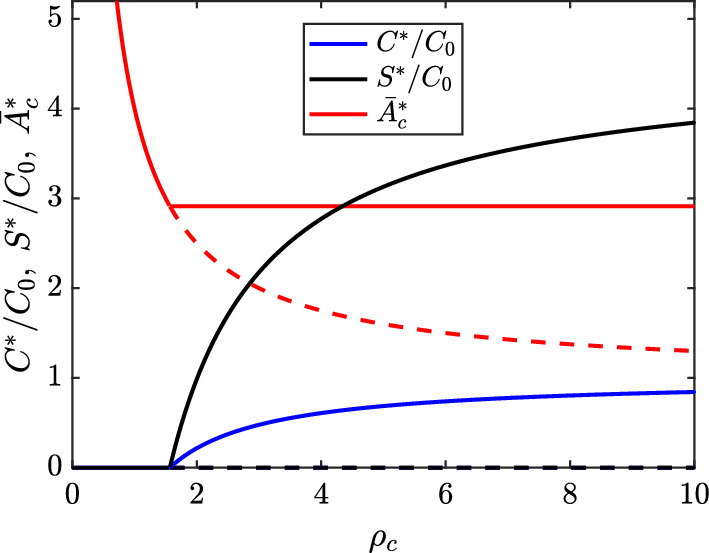
Bifurcation diagram showing the physical steady state solutions (17) and (18) as functions of $\rho _c$. Blue lines show $C^*$, black lines show $S^*$, and red lines show $\bar{A}_c^*$ (both $C^*$ and $S^*$ are normalised by the SMC carrying capacity $C_0$). Stable branches are indicated by solid lines, and unstable branches by dashed lines. We set $\Pi _c = 3$, and the values of all other relevant parameters are given in Table 2

Figure 2 plots the stable and unstable branches of the physical steady state solutions (17) and (18) as functions of the SMC proliferation rate $\rho _c$ for $\Pi _c=3$ and all other parameters at their baseline values. The plot shows that long-term extinction of the SMC (and SDM) population requires $\rho _c<\frac{\Pi _c}{k}\approx 1.568$, while, at the baseline proliferation rate ($\rho _c=8$), the steady state SMC population is relatively close to its theoretical maximum. When $C^*>0$, we find that the ratio of the plotted SMC and SDM population sizes remains constant (i.e., $\frac{S^*}{C^*}=\frac{\frac{\Pi _c}{k} - \beta _c}{\beta _s-\rho _s}\approx 4.558$). This is because the average ingested SMC lipid load *k* is independent of $\rho _c$ for the non-trivial steady state solution (18).

**Fig. 3:**
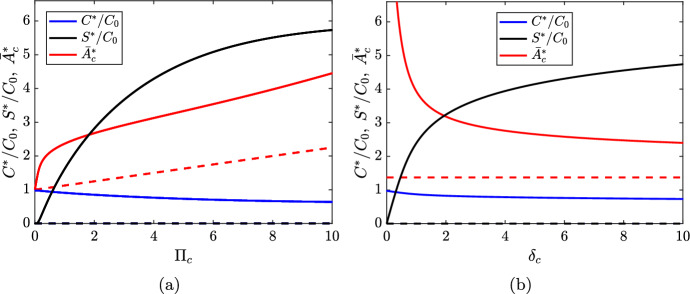
Bifurcation diagrams showing the steady state solutions (17) and (18) as functions of (a) $\Pi _c$ and (b) $\delta _c$. Blue lines show $C^*$, black lines show $S^*$, and red lines show $\bar{A}_c^*$ (both $C^*$ and $S^*$ are normalised by the SMC carrying capacity $C_0$). Stable branches are indicated by solid lines, and unstable branches by dashed lines. In (b), we set $\Pi _c = 3$. Otherwise, all relevant parameters have the values given in Table 2

Figure 3 shows additional bifurcation diagrams for the steady state solutions (17) and (18) as functions of the SMC lipid ingestion rate $\Pi _c$ (Figure 3a) and the maximal phenotypic switching rate $\delta _c$ (Figure 3b). It is noticable in both plots that only relatively small values of these parameters are required for the steady state SDM population size $S^*$ to exceed the steady state SMC population size $C^*$.

Since the condition (20) is *always* satisfied for the baseline parameter values, we find that the nontrivial steady state solution (18) is stable for all $\Pi _c>0$. As $\Pi _c\rightarrow \infty$, the phenotypic switching rate is maximised. In this case, $\frac{C^*_2}{C_0}\rightarrow 1-\frac{\beta _c+\delta _c}{\rho _c}=0.6$, $\bar{A}_{c,2}^*\rightarrow \infty$, and $\frac{S^*_2}{C_0}\rightarrow \left( \frac{\delta _c}{\beta _s - \rho _s}\right) \frac{C^*_2}{C_0}=6$. The approach to this limiting behaviour can be observed in Figure 3a as $\Pi _c$ increases.

Destabilisation of the nontrivial steady state (18) can be achieved by increasing either $\beta _c$ or $\delta _c$. This occurs trivially if $\beta _c>\rho _c$, but the situation for $\delta _c$ is more complicated. Figure 3b indicates that the average SMC lipid load at steady state $\bar{A}_{c,2}^*=k+1$ is a decreasing function of $\delta _c$. This makes sense because an increase in the loss of SMCs to phenotypic switching leads to an increase in SMC proliferation, which in turn reduces average SMC lipid loads (see equation (16b)). As the steady state SMC population size $C_2^*$ is an increasing function of *k*, we find that $C_2^*$ is also a decreasing function of $\delta _c$. For the baseline parameter values with $\Pi _c=3$, $C_2^*$ is seen to decrease very slowly with increasing $\delta _c$ (Figure 3b). The value of $C_2^*$ is observed to remain positive until $\delta _c\approx 6350$, at which point the non-trivial steady state solution (18) exchanges stability with the trivial steady state solution (17). The steady state SDM population $S_2^*$ is found to have a non-monotonic dependence on $\delta _c$ (not shown in Figure 3b). For the baseline parameter values with $\Pi _c=3$, $S_2^*$ is maximised for $\delta _c\approx 220$.

## Results from the Full Model

**Fig. 4:**
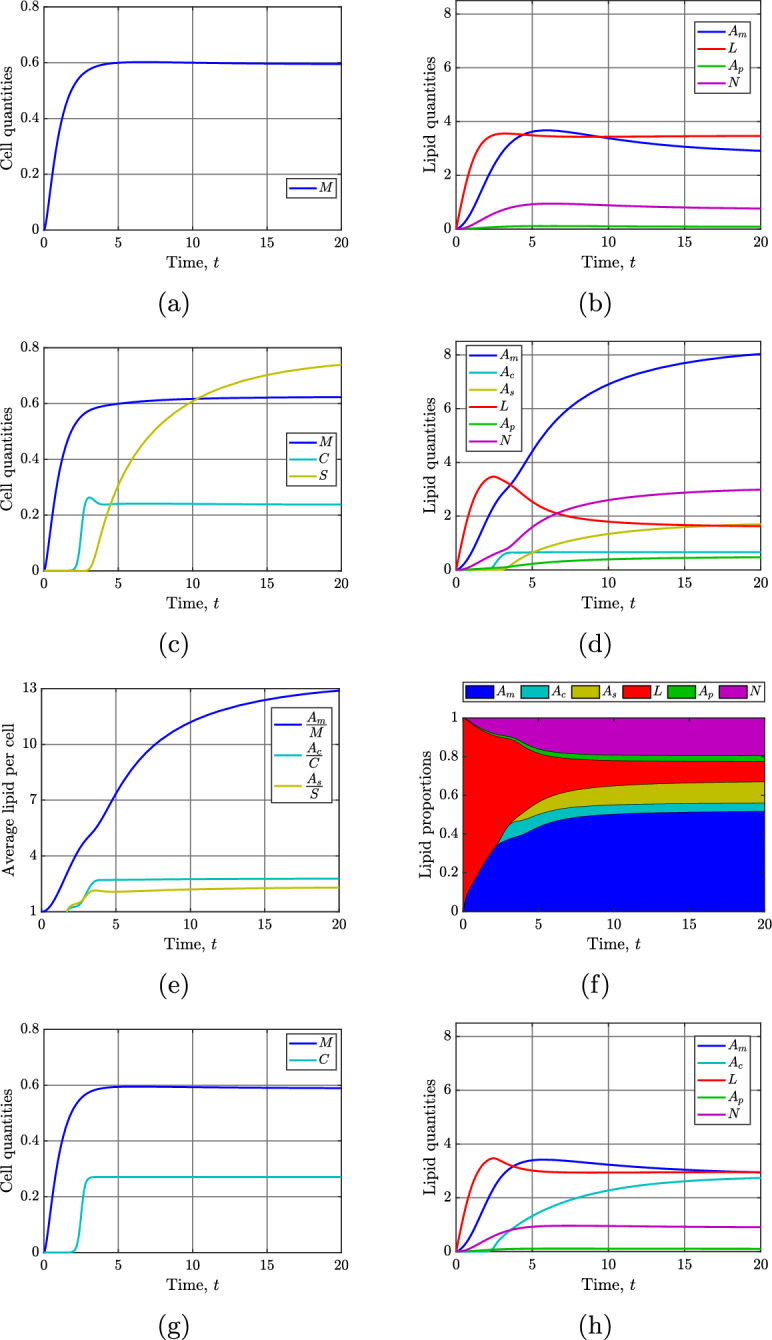
Time-dependent solutions of the model system (13). Panels (a) and (b) show, respectively, the time evolution of the cell and lipid quantities with MDMs only ($C_{init}=0$). Panels (c), (d), (e) and (f), show, respectively, the time evolution of the cell quantities, lipid quantities, average cellular lipid loads, and compartmental lipid proportions with all cell types included. Panels (g) and (h) show, respectively, the time evolution of the cell quantities and lipid quantities with MDMs and SMCs only ($\delta _c=0$). Unless otherwise stated, all parameter values are given in Table 2

Figure 4 shows time-dependent solutions for the atherosclerotic plaque cell and lipid dynamics for the complete model system (13). We first set $C_{init}=0$, and allow the MDM population to evolve in isolation (Figures 4a and 4b). The initial accumulation of extracellular modLDL lipid elicits a near-maximal MDM recruitment response, which is then sustained by the subsequent internalisation of modLDL lipid, endogenous lipids, and lipids from apoptotic and necrotic cells. Eventually, both the MDM numbers and the total MDM lipid load reach a peak before decreasing slightly to equilibrium.

Results for the model with MDMs, SMCs, and SDMs are shown in Figures 4c to 4f. In this simulation, the plaque is seeded with a small initial SMC population $C_{init}=0.0005$ at time $t=t_c=1.7$. The rapid proliferation of these cells quickly drives the SMC population size towards its phenotype switching-free equilibrium (Figure 4c). As the per capita SMC proliferation rate declines, the average SMC lipid load increases (Figure 4e), which drives the phenotypic switching of SMCs to SDMs. The sustained phenotypic switching of SMCs to SDMs causes a small reduction in the SMC population and, in the long-term, leads SDMs to become the dominant cell type in the model plaque (Figure 4c).

Comparing Figure 4d with Figure 4b shows that the inclusion of SMCs and SDMs in the model leads to a greater than 2-fold increase in the total lipid held in the plaque. As the rate of modLDL influx remains constant, and the MDM dynamics are largely unaffected, we conclude that this increase is caused by the substantial *de novo* synthesis of endogenous lipid by the rapidly proliferating SMCs and SDMs. Naturally, some of this additional lipid remains internalised in the SMCs and SDMs. However, we also observe increases in the long-term quantities of MDM lipid, necrotic lipid, and apoptotic lipid (around 3-, 4-, and 5-fold greater than in Figure 4b, respectively). Factors that most likely contribute to these increases are: (1) the absence of SDM (and SMC) emigration out of the plaque, and (2) the relatively large rate of SDM apoptosis. Noticeably, long-term modLDL lipid levels are reduced by around 50% because there are now more cells in the plaque, including SMCs, that can internalise lipid from modLDL.

Figure 4f shows how the proportion of lipid in each model compartment changes over time. Although the proportion of lipid in modLDL is initially decreasing, and the proportion of lipid in MDMs, apoptotic cells and necrotic cells is initially increasing, a noticeable change in the dynamics occurs upon the emergence of SDMs at around $t=3$. As the proportion of lipid in the SDMs grows, there is an acceleration in the growth of the proportions of necrotic, apoptotic, and MDM lipid at the expense of the proportions in SMCs and modLDL.

**Fig. 5:**
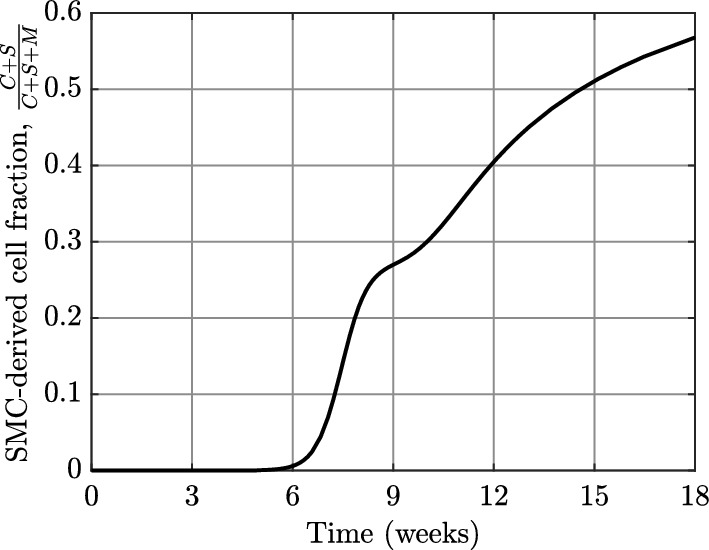
Plot showing the time evolution of the fraction of cells in the model plaque that are derived from the initial SMC population. The curve is plotted against physical time to admit comparison with the experimental data in Figure 2d of Misra et al. (2018)

Figure 5 plots the fraction of SMC-derived cells in the model plaque, $\frac{C+S}{C+S+M}$, against physical time using the data from Figure 4c. Consistent with observations from lineage tracing experiments in mouse models (Misra et al. 2018; Shankman et al. 2015), this plot demonstrates that cells of SMC origin eventually outweigh the proportion of MDMs in the plaque. The temporal evolution of the SMC-derived cell fraction predicted by the model compares favourably with the experimental measurements at weeks 6, 12, and 16 shown in Figure 2b of Misra et al. (2018). While it is encouraging to see the model results align with available experimental data, we emphasise that there may be many alternative model parameterisations capable of producing similar outcomes.

To verify that the increased amounts of apoptotic, necrotic, and MDM lipid seen in Figure 4d are indeed due to the presence of SDMs, we set $\delta _c=0$ to simulate a scenario where SMCs cannot change phenotype (Figures 4g and 4h). In this case, we observe relatively minor increases in the apoptotic, necrotic and MDM lipid. This confirms that it is the emergence of SDMs via phenotype switching that drives the previously observed increase in total lipid in these compartments. Comparing Figures 4h and 4d shows that, in the absence of phenotype switching, the SMC population holds around 4 times more lipid in the long-term. However, in this case, there are no detrimental effects that arise from this increased lipid loading.

**Fig. 6:**
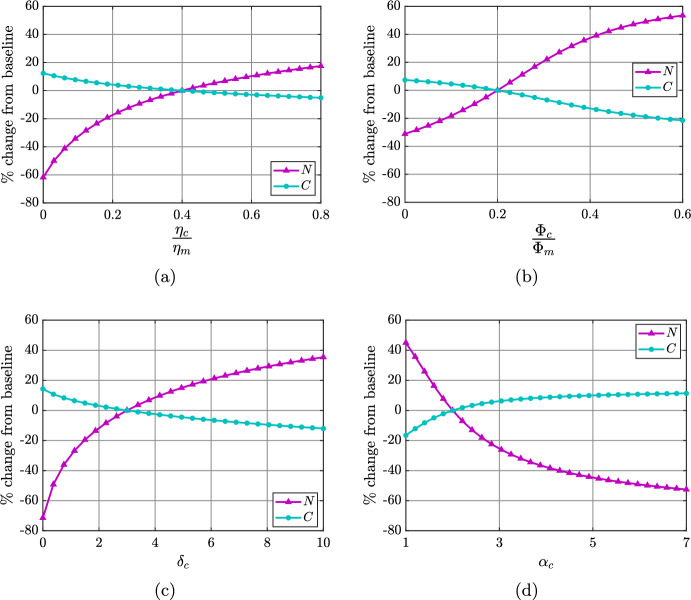
Plots showing the percentage change in steady state SMC numbers (cyan lines) and necrotic lipid mass (magenta lines) in the model plaque as the parameters (a) $\eta _c$, (b) $\Phi _c$, (c) $\delta _c$, and (d) $\alpha _c$ are independently varied from their baseline values

**Fig. 7:**
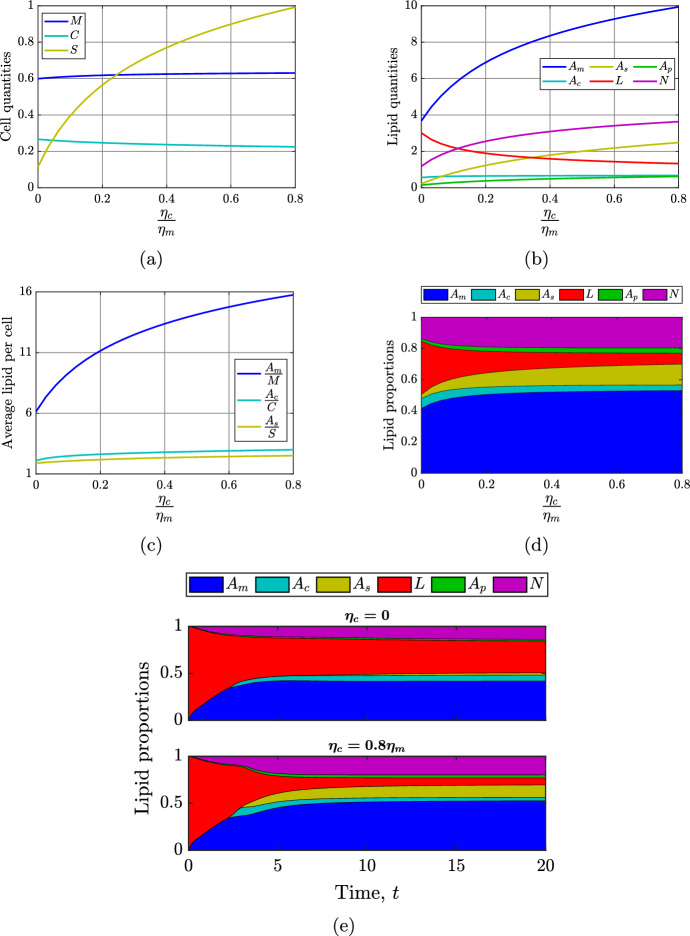
Effect of varying the rate of SMC modLDL consumption over the range $\eta _c\in [0,0.8\eta _m]$. Panels show steady state (a) cell quantities, (b) lipid quantities, (c) average cellular lipid loads, and (d) compartmental lipid proportions as functions of $\eta _c$. Panel (e) shows the time evolution of compartmental lipid proportions for $\eta _c=0$ (top) and $\eta _c=0.8\eta _m$ (bottom). For all simulations, the MDM modLDL consumption rate $\eta _m$ is held at its baseline value

Figure 6 presents plots that show the percentage change in steady state necrotic lipid and cap SMCs as parameters that control SMC lipid consumption ($\eta _c$, $\Phi _c$) and phenotypic switching ($\delta _c$, $\alpha _c$) are varied in the model. As noted in Section 2.6, long-term SMC numbers and necrotic lipid mass can be considered important indicators of the clinical risk associated with a plaque. Figures 7a to 7d present complementary information for the case in which $\eta _c$ is varied (Figure 6a) by showing details of corresponding changes in cell numbers, lipid quantities, average cellular lipid loads, and compartmental lipid proportions.

In Figures 6a and 7a to 7d, the horizontal axis shows the rate of consumption of modLDL by SMCs ($\eta _c$) relative to that by MDMs ($\eta _m$). We observe that as $\eta _c$ increases from 0 to $0.8\eta _m$, SMC numbers reduce by around 15%, while the SDM population increases almost 9-fold (Figure 7a). This is because the phenotypic switch that SMCs undergo is driven by lipid loading, so that if SMCs ingest lipid more rapidly, they are more likely to adopt a macrophage-like phenotype. The MDM population is relatively insensitive to changes in modLDL uptake by SMCs. Thus, as $\eta _c$ increases beyond about $0.25\eta _m$, SDMs become the dominant cell type in the plaque at steady state (Figure 7a).

Figure 7b indicates that, for all $\eta _c\in [0,0.8\eta _m]$, the MDM population is the compartment that carries the most lipid. This lipid load increases around 2.5-fold with $\eta _c$, reflecting an increase in the average cellular lipid load from around 6 to 16 lipid units (Figure 7c). The amount of necrotic lipid at steady state increases by a similar extent to the MDM lipid over the range of $\eta _c$ considered. Predictably, as $\eta _c$ increases, a lower proportion of lipid is in modLDL and a higher proportion is in inside SDMs (Figures 7d and 7e). The proportion of lipid in the other compartments does not change significantly at steady state (Figure 7d), but it must be remembered that there is much more lipid in the system overall when $\eta _c$ is large.

The results in Figures 7d and 7e indicate that, for small $\eta _c$, MDMs are the major lipid-handling cells in the model plaque. However, as $\eta _c$ increases, SDMs bear an increasing lipid burden. Since, in this model, SDMs cannot emigrate out of the plaque, and have limited capacity for lipid export to HDL, an increase in the lipid held in SDMs results in MDMs accumulating more lipid via phagocytosis of apoptotic and necrotic lipid contributed by SDM death. These results suggest that lipid accumulation in SDMs may be linked to an increased likelihood of unstable, lipid-filled plaque formation.

Figure 6b shows the percentage change in steady state SMC numbers and necrotic lipid mass relative to baseline ($\Phi _c=0.2\Phi _m$) as the SMC efferocytosis rate is varied over the range $\Phi _c\in [0,0.6\Phi _m]$. The results are qualitatively similar to those for the case where $\eta _c$ is varied (Figure 6a), showing an increase in necrotic lipid accumulation and a decrease in cap SMC numbers as $\Phi _c$ increases. Figures 6a and 6b therefore confirm that the mechanism of SMC phenotypic switching assumed in this model is independent of the origin of the lipid consumed by SMCs.

The results in Figures 6c and 6d illustrate the impact of varying the maximum SMC phenotypic switching rate $\delta _c$, and the average ingested lipid load for half-maximal SMC switching $\alpha _c$, respectively, on SMC numbers and necrotic lipid at steady state. Consistent with the observations in Figure 4, reducing $\delta _c$ from its baseline value towards zero results in a dramatic reduction in necrotic lipid and a mild increase in SMC numbers. Increasing $\delta _c$ above its baseline value reverses these trends, but the necrotic lipid quantity is less sensitive to changes in $\delta _c$ in this case (Figure 6c). For $\alpha _c$, we observe a trend of increasing SMC numbers and decreasing necrotic lipid as $\alpha _c$ is increased (Figure 6d). This is because increasing $\alpha _c$ suppresses the net phenotypic switching rate of SMCs to SDMs when the average ingested SMC lipid load is small. In practice, this can lead to a temporal delay in the emergence of a SDM population. For example, with $\alpha _c=7$, we find that SDM numbers remain at negligible levels ($<C_{init}$) for approximately 2 weeks of physical time longer than for the baseline case (results not shown).

**Fig. 8:**
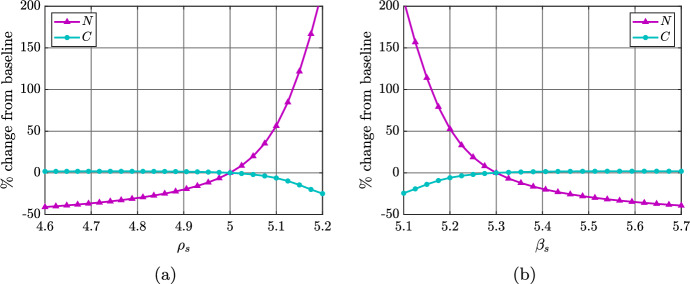
Plots showing the percentage change in steady state SMC numbers (cyan lines) and necrotic lipid mass (magenta lines) in the model plaque as the parameters (a) $\rho _s$, and (b) $\beta _s$ are independently varied from their baseline values

**Fig. 9:**
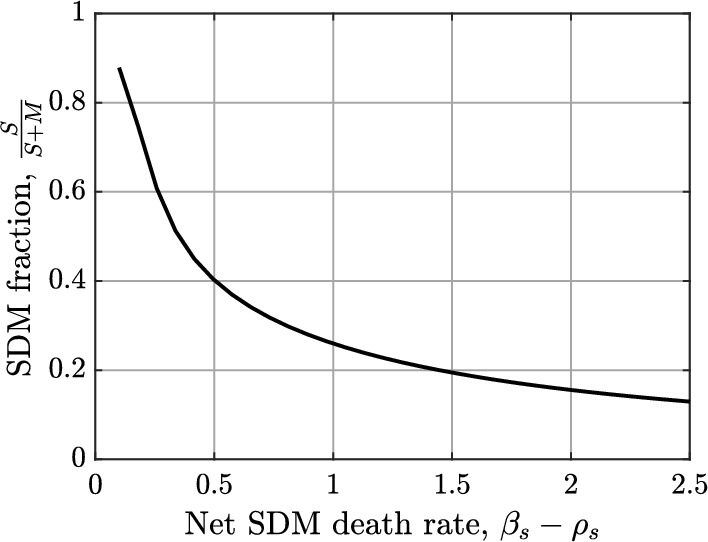
Plot showing the steady state fraction of macrophages that are SDMs as a function of the net SDM death rate, $\beta _s-\rho _s$. For this plot, $\beta _s$ is held at its baseline value, while $\rho _s$ is varied over the range [2.8, 5.2]

**Fig. 10:**
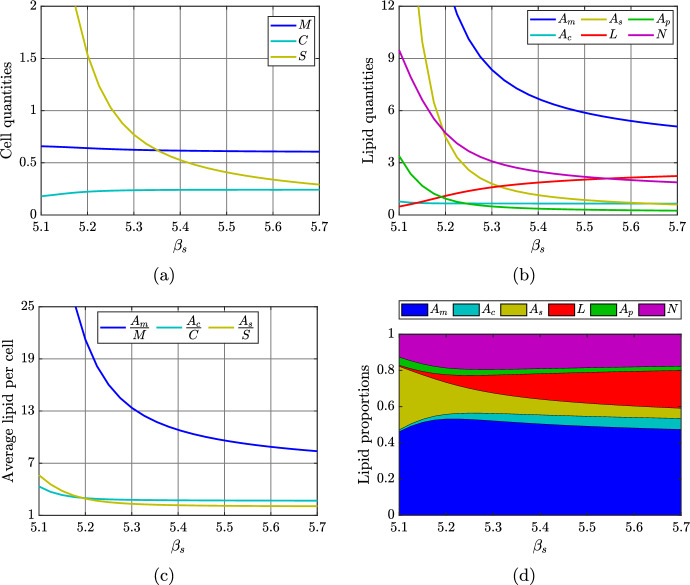
Effect of varying the SDM apoptosis rate over the range $\beta _s\in [5.1,5.7]$. Panels show steady state (a) cell quantities, (b) lipid quantities, (c) average cellular lipid loads, and (d) compartmental lipid proportions as functions of $\beta _s$

Figures 8a and 8b illustrate the percentage changes in steady state SMC numbers and necrotic lipid as the SDM proliferation rate $\rho _s$, and the SDM apoptosis rate $\beta _s$ are independently varied from their baseline values. To ensure that the SDM population does not grow without bound, we consider only values of $\rho _s$ and $\beta _s$ for which the net SDM death rate is positive (i.e., $\beta _s-\rho _s>0$). As $\rho _s$ increases above baseline, or $\beta _s$ decreases below baseline, the steady state necrotic lipid increases dramatically. This is due to a substantial increase in the steady state SDM population (see Figure 10a). The fact that Figures 8a and 8b are almost mirror images implies that the net SDM death rate $\beta _s-\rho _s$ in equation (13e) is the key parameter combination that underlies these results. This is despite the fact that $\rho _s$ and $\beta _s$ appear independently in equation (13f). As the net SDM death rate approaches zero, there is a rapid increase in the proportion of plaque macrophages, at steady state, that are SDMs (Figure 9). This corresponds to a highly pathological plaque state.

A more detailed illustration of the effects of changing the SDM apoptosis rate $\beta _s$ (for fixed $\rho _s$) is given in Figure 10. For $\beta _s$ close to $\rho _s$ (small net death rate), the dramatic increase in steady state SDM numbers is accompanied by a dramatic increase in the amount of lipid held in both the SDM and MDM populations (Figure 10b). However, the average lipid per cell in MDMs at steady state is much higher than the average lipid per cell in SDMs (Figure 10c), and MDMs carry a larger proportion of the plaque intracellular lipid (Figure 10d). As well as presumably contributing to substantial plaque growth, our results suggest that a small net SDM death rate may lead to increased plaque inflammation as heavily lipid-laden MDMs are likely to release more inflammatory cytokines.

**Fig. 11:**
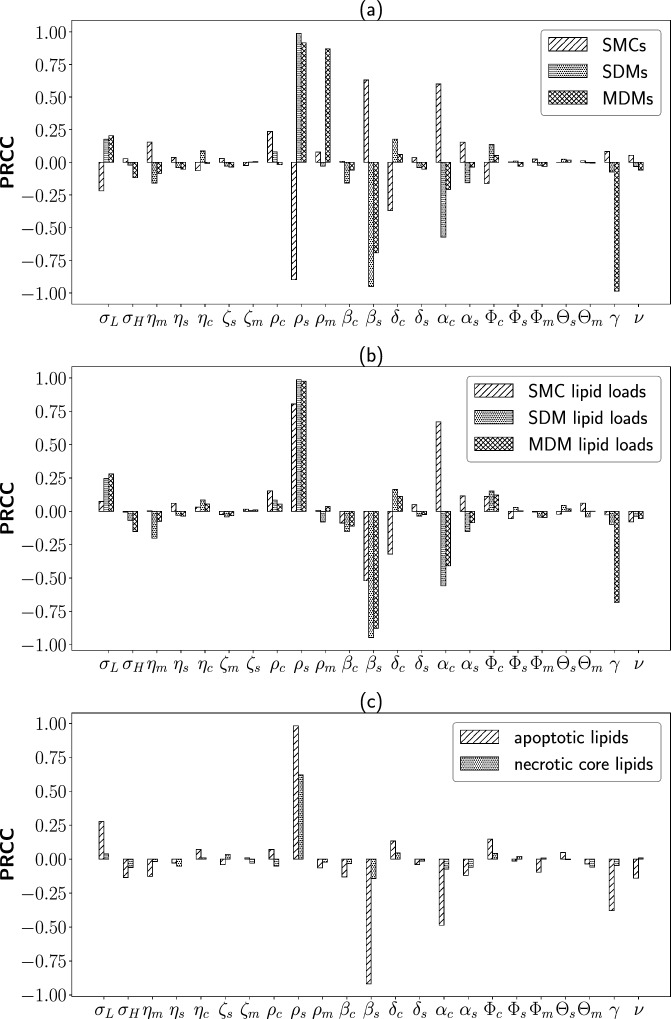
Results of a parameter sensitivity analysis on the model (13) showing PRCC values for each model parameter with respect to steady state (a) cell quantities, (b) intracellular lipid quantities, and (c) apoptotic and necrotic lipid quantities. Parameters were varied by $\pm 20\%$ from their baseline values, and the baseline parameter values used in this analysis are listed in Table 2. Samples violating the constraint $\rho _s\le \beta _s$ were excluded from the analysis to avoid scenarios with unrealistic growth of the SDM population

To comprehensively study the effect of varying parameter values away from baseline on the steady state solutions of the model, we used Latin hypercube sampling and calculated partial rank correlation coefficients (PRCCs) (Blower and Dowlatabadi 1994; Marino et al. 2008) with 1000 simulations per run. The results of this sensitivity analysis are shown in Figure 11. For a given model variable and parameter, a PRCC value close to $+1$ or $-1$ indicates a strong positive or negative correlation, respectively, between the parameter and the value of the variable at steady state. Positive PRCC values indicate that the value of the variable increases as the value of the parameter increases, while negative PRCC values indicate that the value of the variable decreases as the value of the parameter increases. Figure 11 shows that the MDM emigration rate, and the MDM and SDM proliferation and apoptosis rates ($\gamma$, $\rho _m$, $\rho _s$, $\beta _s$) clearly have the most impact on cell populations. (Recall that the MDM apoptosis rate $\beta _m$ has been scaled to 1 in the dimensionless system (13), so is not explicitly varied in this analysis). The values $\rho _s$ and $\beta _s$ also have a significant impact on the intracellular lipids, and the apoptotic and necrotic lipids, as shown above. Interestingly, the parameter related to phenotypic switching that has the greatest impact is $\alpha _c$, which defines the average ingested lipid load for a half-maximal SMC switching rate. In addition to the expected influence on steady state SMC and SDM populations, $\alpha _c$ also strongly impacts the lipid held in each of the three cell types as well as in apoptotic cells.

## Discussion

This paper presents an ODE model of SMC phenotypic switching in the atherosclerotic plaque. We use the model to explore the impact of SMC phenotypic switching on plaque progression, with a focus on cell dynamics and lipid accumulation. The model assumes that plaque cells can ingest lipid from modified LDL particles and dead cells, and that plaque cells can efflux lipid to HDL particles. The model also includes recruitment of MDMs, emigration of MDMs, and proliferation and death of all plaque cell types. We explore how SMC phenotypic switching into macrophage-like cells (SDMs) can drive pathological plaque formation by increasing necrotic core lipid and depleting the fibrous cap SMC population.

The modelling assumptions incorporate several significant observations from recent experimental studies. For example, the murine plaque lineage tracing study by Misra et al. (2018) showed that the plaque SMC population is initiated by one or two highly proliferative progenitor cells that enter the plaque after 5–6 weeks of feeding on a high-fat diet. This is modelled by assuming that the SMC population is zero for the first 5 weeks of physical time before a very small number of rapidly proliferating SMCs is added to the plaque to initiate population growth. The *in vitro* study by Vengrenyuk et al. (2015) showed that cholesterol loading of vascular SMCs leads to reduced expression of typical SMC markers and increased expression of typical macrophage markers. Vengrenyuk et al. (2015) further showed that these trends of marker expression can be reversed if SMCs can offload their internalised lipid. These observations are modelled by assuming bidirectional phenotypic switching between SMCs and SDMs, where the respective switching rates are given by appropriately defined functions of internalised cellular lipid loads.

The full mathematical model (13), which comprises ten coupled nonlinear ODEs, is naturally complicated because many players contribute to lipid trafficking in mature plaques. However, under some simplifying assumptions, we find that the dynamics of SMC phenotypic switching can be well captured by a three-equation submodel (15) that decouples from the full system. Steady state analysis of this reduced model provides insight into the factors that determine the long-term size of the model SDM population. This insight is valuable because numerical simulations of the full model suggest that SDM population growth is a key driver of model plaque pathology. The results in Figures  7 and 10, for example, show that an increase in the long-term SDM population size leads to a concurrent increase in the lipid held in the necrotic core.

Steady state analysis of the reduced model reveals that, in the absence of SDM-to-SMC restoration ($\delta _s=0$), the fates available for the SMC-derived populations are co-existence (18) and extinction (17). For long-term coexistence of SMCs and SDMs in the model plaque, the rate of SMC proliferation must exceed the rate of SMC loss due to the combined effects of apoptosis and phenotypic switching. It is perhaps surprising to find that excessive phenotypic switching of SMCs can cause extinction of *both* cell populations, but this is because we assume that SDM apoptosis exceeds SDM proliferation ($\beta _s>\rho _s$). In the alternative case where $\rho _s>\beta _s$, extinction of the SMC population remains possible, but the SDM population always grows without bound. The potential for unbounded SDM population growth could be removed from the model by introducing a bounded, nonlinear (e.g., logistic) term for SDM proliferation. However, we chose not to pursue this in the current study because: (1) any choice of limiting population size would be arbitrary; and (2) the SDM lipid equation (13f) would become ambiguous if this limiting size was exceeded.

The steady state analysis of the reduced model is valid and interesting in itself. However, it is natural to consider whether this analysis remains valid in the context of the full model. The numerical steady state solutions presented in Section 4 provide evidence that it does. As $\beta _s$ is reduced towards $\rho _s$ in Figure 10, for example, we see a substantial increase in the SDM population (Figure 10a), and minimal variation in both the SMC population (Figure 10a) and the SMC average lipid load (Figure 10c). This is consistent with the steady state solution (18), in which $S_2^*$ is inversely proportional to $\beta _s-\rho _s$, and both $C_2^*$ and $\bar{A}_{c,2}^*$ are independent of $\beta _s$. Consistency between analytical and numerical results can also be seen in Figure 7. As the SMC modLDL ingestion rate $\eta _c$ is increased, the qualitative trends in the steady state values of *C* (Figure 7a), $\bar{A}_c$ (Figure 7c), and *S* (Figure 7a) are the same as those depicted in Figure 3a for an increasing SMC lipid ingestion rate $\Pi _c$. In Appendix A we provide an alternative perspective on the consistency between outcomes for the reduced model and the full model. Using the baseline parameter values as a reference case, we demonstrate the validity of the simplifying assumptions that are used to derive the reduced model.

Results from numerical simulations of the full model collectively highlight the critical role of SMCs in lipid trafficking within the plaque. When SMCs ingest sufficient lipid from modLDL or apoptotic cells, they transition to SDMs, which ultimately contribute to expansion of the plaque necrotic core. Figure 4d, for example, shows a 4-fold increase in necrotic lipid compared to an equivalent case where no SMCs enter the model plaque (Figure 4b). The loss of functional SMCs due to phenotypic switching also reduces the cap SMC population, which then relies upon rapid and sustained SMC proliferation to prevent excessive depletion of these critical cells. The necrotic lipid accumulation and cap SMC loss that we observe in the presence of SMC phenotypic switching is indicative of heightened plaque vulnerability. The model therefore suggests that inhibiting SMC-to-SDM phenotypic switching, by targeting processes such as SMC lipid consumption, could help to reduce plaque vulnerability.

The full model leads us to the following conclusions about the impact on plaque fate of SMCs switching phenotype to become macrophage-like SDMs.

- The total number of plaque cells with macrophage phenotype can increase significantly when SMCs switch to SDMs. When the net SDM death rate is small, this can result in dramatically more macrophage-like cells in the plaque. This result is in agreement with results from experiments in mice, where observations show that large and growing plaques may contain significant populations of SDMs (Pan et al. 2024).
- In the model, MDM numbers are not significantly altered in the presence of SDMs. However, the average MDM lipid load is significantly increased in the presence of SDMs. Several factors contribute to this phenomenon. First, the rapid and unfettered proliferation of SDMs increases the overall plaque lipid content via the *de novo* endogenous lipid synthesis required to form new daughter cells. Second, it is assumed that SDMs cannot offload internalised lipid to HDL as efficiently as MDMs (Francis 2023), nor emigrate out of the plaque taking their internalised lipid with them. Hence, the only fate available to SDMs is to die in the plaque. When an SDM dies, its internalised lipid is added to the apoptotic lipid pool. This lipid is mainly efferocytosed by MDMs, whose efferocytosis rate in the model is four times that of SDMs and five times that of SMCs.
- The average SDM lipid load in the model is considerably smaller than the average lipid load of MDMs. This observation reflects differences between the population dynamics and lipid trafficking properties of the two cell species. SDMs have substantially higher proliferation and death rates compared to MDMs. This means that the lipid in SDMs is frequently divided between daughter cells that ultimately have short lifespans with limited time for lipid consumption. Moreover, SDMs consume all types of lipid at a slower rate than MDMs. Hence, even though the SDM population may be large, a very significant proportion of plaque internalised lipid is still held by MDMs. If our assumptions are valid, we would expect that most of the heavily lipid laden cells in a plaque are MDMs and not SDMs.

The theoretical work presented in this paper is encouraging because the findings align with the prevailing hypothesis that SDMs can exacerbate plaque progression (Shankman et al. 2015). Moreover, there are several interesting emergent phenomena in the results. We are hesitant, however, to label the model as “predictive”. Rather, we consider that the model identifies potential avenues for future experimental investigation that could ultimately validate (or otherwise) the current modelling assumptions.

The model contains a large number of parameters. While comprehensive efforts have been made to obtain accurate estimates (see Table 1 and Figure 5) and identify the parameters that most influence plaque fate (Figure 11), we have not explicitly quantified the uncertainty in our estimates nor identified correlations between individual values. We consider this to be an important target for future work.

We have performed a preliminary structural identifiability analysis of the model using the Julia package *StructuralIdentifiability.jl* (Dong et al. 2023). This analysis aims to ascertain whether the model parameters could be recovered if constrained by perfect (i.e., continuous and noise-free) data on a given set of model outputs. Making the conservative assumption that only the cell population sizes (*M*(*t*), *C*(*t*), *S*(*t*)) are observable, we find that *StructuralIdentifiability.jl* fails to produce an output for the full model. This suggests that the model may be too large and complex to perform the necessary algebraic computation. Reduction techniques such as generalised first integrals (Liyanage et al. 2026) may therefore be required to simplify the system.

Repeating the above analysis for the reduced model, we find that, with known initial conditions, almost all parameters are globally identifiable. The only exceptions are: (1) $\rho _s$ and $\beta _s$ which are globally identifiable only as the combination $(\rho _s-\beta _s)$; and (2) $\alpha _c$, which is locally identifiable. This preliminary analysis builds confidence that the reduced model is amenable to parameter estimation and uncertainty quantification via practical identifiability analysis. Of course, real experimental data (such as that in Misra et al. (2018)) is neither continuous nor noise-free, which may prove to be restrictive. Moreover, even if data can be used to accurately parameterise the model, this does not rule out the possibility that modelling choices such as the functional forms for the phenotypic switching rates are misspecified (Browning et al. 2026).

The model reported in this work has some limitations to be addressed in future studies. For example, the ODE formulation of the model means that the phenotypic switching rates are expressed in terms of the average ingested lipid loads across the entire SMC and SDM populations. In reality, the cells within these populations will have a spectrum of ingested lipid loads, and only a particular subset of cells would be likely to undergo phenotype change at any given time. This limitation could be addressed by formulating the model with resolution in cellular lipid loads (Ford et al. 2019b; Chambers et al. 2024). In such a framework, the lipid-dependent cell behaviour could also be extended to include MDMs. Here, we assume that MDMs can acquire very large lipid loads without any loss of normal function. If MDM function were assumed to be impaired by lipid loading (Chambers et al. 2023; Watson et al. 2023), it is possible that the implications of SMC phenotypic switching for plaque fate could be considerably worse than predicted in this study.

A further interesting target for future studies is to formulate the model with resolution in space. This could lead to a more comprehensive understanding of exactly how cap SMCs are exposed to lipid in the plaque, and how the movement of plaque cells (e.g., SDMs vacating the cap region) influence the long-term fate and spatial structure of the plaque. An intriguing possibility would be to combine spatial structure with lipid structure, as reported in a recent model for macrophages in the early human plaque (Chambers et al. 2025).

The model proposed in this paper is informed by observations of plaque SMC behaviour in murine models of atherosclerosis, and we therefore urge some caution in interpreting our findings with respect to the human condition. We assume, for example, that the plaque is initially devoid of SMCs until they enter the plaque as cap-forming cells (Misra et al. 2018). This particular assumption is not consistent with human atherosclerosis because the human intima contains a resident SMC population that is present prior to plaque growth. Despite this difference, experimental studies have shown consistency of human pathology with mouse models in many aspects of plaque SMC behaviour, including in phenotypic switching to SDMs (Misra et al. 2018; Shankman et al. 2015). Thus, we anticipate that our findings should at least be qualitatively conserved in the case of human atherosclerosis, even if not all SMCs that differentiate into SDMs contribute to depletion of the fibrous cap.

This model for vascular SMC phenotypic switching in atherosclerosis highlights the crucial role of SMC plasticity on the dynamics of cells and lipids during atherosclerotic plaque progression. The reported results offer potentially useful insights that may contribute to the future development of therapeutic strategies aimed at stabilising vulnerable plaques and mitigating adverse cardiovascular outcomes.

## Figures and Tables

**Fig. 12:**
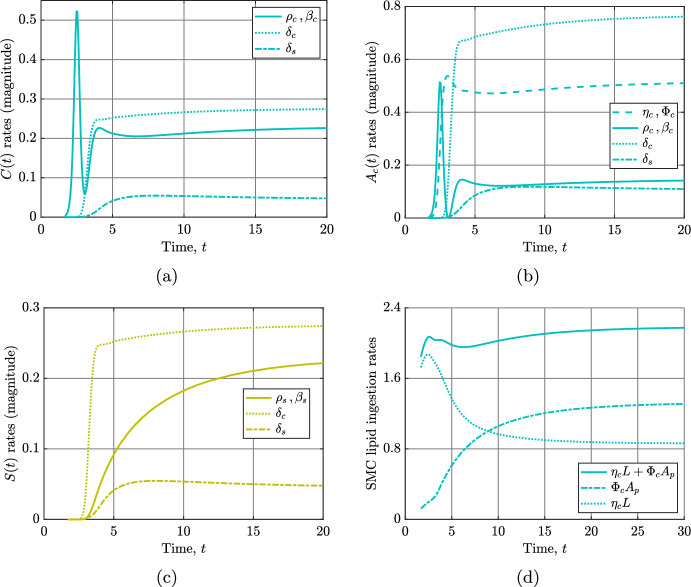
Evidence for the validity of the model reduction in Section 3. Plots show the time evolution of (a–c) magnitudes of terms in the ODEs for *C*(*t*), $A_c(t)$, and *S*(*t*), respectively, and (d) lipid ingestion rates per SMC, for the system (13) with parameter values in Table 2. In (a–c), parameters in legend entries denote which terms are plotted on each line. A legend entry with two parameters indicates that the contributions of two individual terms have been combined. In (d), plots show the total rate of lipid ingestion ($\eta _c L+\Phi _c A_p$), the rate of modLDL lipid ingestion ($\eta _c L$), and the rate of apoptotic lipid ingestion ($\Phi _c A_p$)

## Data Availability

Data sharing not applicable to this article as no datasets were generated or analysed during the current study.

## Validity of Model Reduction

The reduced model (15) that we analyse in Section 3 is derived from the full model (13) via two simplifying assumptions. First, that SDM-to-SMC phenotypic switching is negligible (i.e., $\delta _s=0$); and, second, that the total rate of lipid ingestion per SMC is constant (i.e., $\eta _c L(t)+\Phi _c A_p(t)\approx \Pi _c$). Figure 12 plots additional data from the base case simulation (Figures 4c–4f) to show that these assumptions can be valid. Figures 12a, 12b, and 12c plot the time evolution of magnitudes of terms in the ODEs for *C*(*t*), $A_c(t)$, and *S*(*t*), respectively. These plots show that the contributions of terms in $\delta _s$ remain small relative to other terms in the equations for all time. Figure 12d shows that the total rate of lipid ingestion per SMC ($\eta _c L+\Phi _c A_p$) has only mild temporal variation during the simulation. This is because, while the rate of modLDL lipid ingestion ($\eta _c L$) decreases, there is a compensatory increase in the rate of apoptotic lipid ingestion ($\Phi _c A_p$). These plots suggest that the reduced model of SMC phenotypic switching (15) can be sufficient to encapsulate the key features of SMC phenotypic switching in the full model (13). This implies that the results from the steady state analysis in Section 3 can approximately hold for the full model.

## References

[CR1] Allahverdian S, Pannu PS, Francis GA (2012) Contribution of monocyte-derived macrophages and smooth muscle cells to arterial foam cell formation. Cardiovasc Res 95(2):165–172 22345306 10.1093/cvr/cvs094

[CR2] Allahverdian S, Chehroudi AC, McManus BM, Abraham T, Francis GA (2014) Contribution of intimal smooth muscle cells to cholesterol accumulation and macrophage-like cells in human atherosclerosis. Circulation 129(15):1551–1559 24481950 10.1161/CIRCULATIONAHA.113.005015

[CR3] Allahverdian S, Chaabane C, Boukais K, Francis GA, Bochaton-Piallat ML (2018) Smooth muscle cell fate and plasticity in atherosclerosis. Cardiovasc Res 114(4):540–550 29385543 10.1093/cvr/cvy022 PMC5852505

[CR4] Bäck M, Yurdagul A Jr, Tabas I, Öörni K, Kovanen PT (2019) Inflammation and its resolution in atherosclerosis: mediators and therapeutic opportunities. Nat Rev Cardiol 16(7):389–406 30846875 10.1038/s41569-019-0169-2 PMC6727648

[CR5] Bennett MR, Sinha S, Owens GK (2016) Vascular smooth muscle cells in atherosclerosis. Circ Res 118(4):692–702 26892967 10.1161/CIRCRESAHA.115.306361 PMC4762053

[CR6] Beyea MM, Reaume S, Sawyez CG, Edwards JY, O’Neil C, Hegele RA, Pickering JG, Huff MW (2012) The oxysterol 24(S), 25-epoxycholesterol attenuates human smooth muscle-derived foam cell formation via reduced low-density lipoprotein uptake and enhanced cholesterol efflux. J Am Heart Assoc 1(3):000810 10.1161/JAHA.112.000810 PMC3487330 23130136

[CR7] Blower SM, Dowlatabadi H (1994) Sensitivity and uncertainty analysis of complex models of disease transmission: an HIV model, as an example. Int Stat Rev 62(2):229–243

[CR8] Brown MS, Goldstein JL (1983) Lipoprotein metabolism in the macrophage: implications for cholesterol deposition in atherosclerosis. Annu Rev Biochem 52(1):223–261 6311077 10.1146/annurev.bi.52.070183.001255

[CR9] Browning AP, Flegg JA, Murphy RJ (2026) A cautionary tale of model misspecification and identifiability. Bull Math Biol 88:5 10.1007/s11538-025-01573-4 41413679

[CR10] Bulelzai MAK, Dubbeldam JLA, Meijer HGE (2014) Bifurcation analysis of a model for atherosclerotic plaque evolution. Physica D 278:31–43

[CR11] Cai C, Zhu H, Ning X, Li L, Yang B, Chen S, Wang L, Lu X, Gu D (2019) LncRNA ENST00000602558.1 regulates ABCG1 expression and cholesterol efflux from vascular smooth muscle cells through a p65-dependent pathway. Atherosclerosis 285:31–39 31003090 10.1016/j.atherosclerosis.2019.04.204

[CR12] Casula M, Colpani O, Xie S, Catapano AL, Baragetti A (2021) HDL in atherosclerotic cardiovascular disease: in search of a role. Cells 10(8):1869 34440638 10.3390/cells10081869 PMC8394469

[CR13] Chambers KL, Myerscough MR, Byrne HM (2023) A new lipid-structured model to investigate the opposing effects of LDL and HDL on atherosclerotic plaque macrophages. Math Biosci 357:108971 36716850 10.1016/j.mbs.2023.108971

[CR14] Chambers KL, Watson MG, Myerscough MR (2024) A lipid-structured model of atherosclerosis with macrophage proliferation. Bull Math Biol 86(8):104 38980556 10.1007/s11538-024-01333-w PMC11233351

[CR15] Chambers KL, Myerscough MR, Watson MG, Byrne HM (2025) A spatially resolved and lipid-structured model for macrophage populations in early human atherosclerotic lesions. J Theor Biol 614:112232 40763824 10.1016/j.jtbi.2025.112232

[CR16] Cohen A, Myerscough MR, Thompson RS (2014) Athero-protective effects of high density lipoproteins (HDL): an ODE model of the early stages of atherosclerosis. Bull Math Biol 76:1117–1142 24722888 10.1007/s11538-014-9948-4

[CR17] Cooper G, Adams K (2022) The cell: a molecular approach. Oxford University Press USA

[CR18] Dong R, Goodbrake C, Harrington H, Pogudin G (2023) Differential elimination for dynamical models via projections with applications to structural identifiability. SIAGA 7(1):194–235

[CR19] El Khatib N, Génieys S, Kazmierczak B, Volpert V (2009) Mathematical modelling of atherosclerosis as an inflammatory disease. Phil Trans R Soc A 367(1908):4877–4886 19884184 10.1098/rsta.2009.0142

[CR20] Ford HZ, Zeboudj L, Purvis GSD, Ten Bokum A, Zarebski AE, Bull JA, Byrne HM, Myerscough MR, Greaves DR (2019) Efferocytosis perpetuates substance accumulation inside macrophage populations. Proc R Soc B 286(1904):20190730 10.1098/rspb.2019.0730 PMC6571464 31161905

[CR21] Ford HZ, Byrne HM, Myerscough MR (2019) A lipid-structured model for macrophage populations in atherosclerotic plaques. J Theor Biol 479:48–63 31319051 10.1016/j.jtbi.2019.07.003

[CR22] Francis GA (2023) The greatly under-represented role of smooth muscle cells in atherosclerosis. Curr Atheroscler Rep 25(10):741–749 37665492 10.1007/s11883-023-01145-8 PMC10564813

[CR23] Gisterå A, Hansson GK (2017) The immunology of atherosclerosis. Nature Rev Nephrol 13(6):368–380 28392564 10.1038/nrneph.2017.51

[CR24] Goldberg D, Khatib S (2022) Atherogenesis, transcytosis, and the transmural cholesterol flux: a critical review. Oxid Med Cell Longev 2022:2253478 35464770 10.1155/2022/2253478 PMC9023196

[CR25] Gomez D, Owens GK (2012) Smooth muscle cell phenotypic switching in atherosclerosis. Cardiovasc Res 95(2):156–164 22406749 10.1093/cvr/cvs115 PMC3388816

[CR26] Hansson GK (2005) Inflammation, atherosclerosis, and coronary artery disease. N Engl J Med 352(16):1685–1695 15843671 10.1056/NEJMra043430

[CR27] Hansson GK, Hermansson A (2011) The immune system in atherosclerosis. Nat Immunol 12(3):204–212 21321594 10.1038/ni.2001

[CR28] Harrington JR (2000) The role of MCP-1 in atherosclerosis. Stem Cells 18(1):65–66 10661575 10.1634/stemcells.18-1-65

[CR29] Hedin U, Roy J, Tran PK (2004) Control of smooth muscle cell proliferation in vascular disease. Curr Opin Lipidol 15(5):559–565 15361792 10.1097/00041433-200410000-00010

[CR30] Kim KW, Ivanov S, Williams JW (2020) Monocyte recruitment, specification, and function in atherosclerosis. Cells 10(1):15 33374145 10.3390/cells10010015 PMC7823291

[CR31] Kontush A, Lindahl M, Lhomme M, Calabresi L, Chapman MJ, Davidson WS (2015) Structure of HDL: particle subclasses and molecular components. In: Eckardstein A, Kardassis D (eds) High density lipoproteins: from biological understanding to clinical exploitation, pp 3–51. Springer 10.1007/978-3-319-09665-0_1 25522985

[CR32] Lee JG, Koh SJ, Yoo SY, Yu JR, Lee SA, Koh G, Lee D (2012) Characteristics of subjects with very low serum low-density lipoprotein cholesterol and the risk for intracerebral hemorrhage. Korean J Intern Med 27(3):317 23019397 10.3904/kjim.2012.27.3.317 PMC3443725

[CR33] Lee SJ, Baek SE, Jang MA, Kim CD (2019) SIRT1 inhibits monocyte adhesion to the vascular endothelium by suppressing Mac-1 expression on monocytes. Exp Mol Med 51(4):1–12 10.1038/s12276-019-0239-x PMC6483987 31023999

[CR34] Lhoták Š, Gyulay G, Cutz J-C, Al-Hashimi A, Trigatti BL, Richards CD, Igdoura SA, Steinberg GR, Bramson J, Ask K, Austin RC (2016) Characterization of proliferating lesion-resident cells during all stages of atherosclerotic growth. J Am Heart Assoc 5:003945 10.1161/JAHA.116.003945 PMC5015311 27528409

[CR35] Libby P (2002) Atherosclerosis: the new view. Sci Am 286(5):46–55 10.1038/scientificamerican0502-46 11951331

[CR36] Liu YX, Yuan PZ, Wu JH, Hu B (2021) Lipid accumulation and novel insight into vascular smooth muscle cells in atherosclerosis. J Mol Med 99(11):1511–1526 34345929 10.1007/s00109-021-02109-8

[CR37] Liyanage YR, Saucedo O, Tuncer N, Chowell G (2026) A Tutorial on Structural Identifiability of Epidemic Models Using StructuralIdentifiability.jl. https://arxiv.org/abs/2505.10517 10.1007/s11538-026-01648-w 42234202

[CR38] Marino S, Hogue IB, Ray CJ, Kirschner DE (2008) A methodology for performing global uncertainty and sensitivity analysis in systems biology. J Theor Biol 254(1):178–196 18572196 10.1016/j.jtbi.2008.04.011 PMC2570191

[CR39] Matyus SP, Braun PJ, Wolak-Dinsmore J, Saenger AK, Jeyarajah EJ, Shalaurova I, Warner SM, Fischer TJ, Connelly MA (2015) HDL particle number measured on the Vantera®, the first clinical NMR analyzer. Clin Biochem 48(3):148–155 25438074 10.1016/j.clinbiochem.2014.11.017

[CR40] Mehrhof FB, Schmidt-Ullrich R, Dietz R, Scheidereit C (2005) Regulation of vascular smooth muscle cell proliferation: role of NF-B revisited. Circ Res 96(9):958–964 15831813 10.1161/01.RES.0000166924.31219.49

[CR41] Misra A, Feng Z, Chandran RR, Kabir I, Rotllan N, Aryal B, Sheikh AQ, Ding L, Qin L, Fernández-Hernando C, Tellides G, Greif DM (2018) Integrin beta3 regulates clonality and fate of smooth muscle-derived atherosclerotic plaque cells. Nat Commun 9(1):2073 29802249 10.1038/s41467-018-04447-7 PMC5970166

[CR42] Nielsen LB (1996) Transfer of low density lipoprotein into the arterial wall and risk of atherosclerosis. Atherosclerosis 123(1–2):1–15 8782833 10.1016/0021-9150(96)05802-9

[CR43] Orlova EV, Sherman MB, Chiu W, Mowri H, Smith LC, Gotto AM Jr (1999) Three-dimensional structure of low density lipoproteins by electron cryomicroscopy. Proc Nat Acad Sci U S A 96(15):8420–8425 10.1073/pnas.96.15.8420 PMC17531 10411890

[CR44] Ougrinovskaia A, Thompson RS, Myerscough MR (2010) An ODE model of early stages of atherosclerosis: mechanisms of the inflammatory response. Bull Math Biol 72:1534–1561 20440571 10.1007/s11538-010-9509-4

[CR45] Pan J, Cai Y, Liu M, Li Z (2021) Role of vascular smooth muscle cell phenotypic switching in plaque progression: a hybrid modeling study. J Theor Biol 526:110794 34087268 10.1016/j.jtbi.2021.110794

[CR46] Pan H, Ho SE, Xue C, Cui J, Johanson QS, Sachs N, Ross LS, Li F, Solomon RA, Connolly ES, Patel VI, Maegdefessel L, Zhang H, Reilly MP (2024) Atherosclerosis is a smooth muscle cell-driven tumor-like disease. Circulation 149(24):1885–1898 38686559 10.1161/CIRCULATIONAHA.123.067587 PMC11164647

[CR47] Randolph GJ (2008) Emigration of monocyte-derived cells to lymph nodes during resolution of inflammation and its failure in atherosclerosis. Curr Opin Lipidol 19(5):462–468 18769227 10.1097/MOL.0b013e32830d5f09 PMC2652166

[CR48] Reape TJ, Groot PH (1999) Chemokines and atherosclerosis. Atherosclerosis 147(2):213–225 10559506 10.1016/s0021-9150(99)00346-9

[CR49] Robbins CS, Hilgendorf I, Weber GF, Theurl I, Iwamoto Y, Figueiredo J, Gorbatov R, Sukhova GK, Gerhardt LMS, Smyth D, Zavitz CCJ, Shikatani EA, Parsons M, Rooijen N, Lin HY, Husain M, Libby P, Nahrendorf M, Weissleder R, Swirski FK (2013) Local proliferation dominates lesional macrophage accumulation in atherosclerosis. Nat Med 19(9):1166–1172 23933982 10.1038/nm.3258 PMC3769444

[CR50] Rodriguez Sawicki L, Garcia KA, Corsico B, Scaglia N (2019) De novo lipogenesis at the mitotic exit is used for nuclear envelope reassembly/expansion. Implic Comb Chemother Cell Cycle 18(14):1646–1659 10.1080/15384101.2019.1629792 PMC6619966 31203714

[CR51] Saraste A, Pulkki K (2000) Morphologic and biochemical hallmarks of apoptosis. Cardiovasc Res 45(3):528–537 10728374 10.1016/s0008-6363(99)00384-3

[CR52] Scaglia N, Tyekucheva S, Zadra G, Photopoulos C, Loda M (2014) De novo fatty acid synthesis at the mitotic exit is required to complete cellular division. Cell Cycle 13(5):859–868 24418822 10.4161/cc.27767 PMC3979921

[CR53] Schrijvers DM, De Meyer GRY, Kockx MM, Herman AG, Martinet W (2005) Phagocytosis of apoptotic cells by macrophages is impaired in atherosclerosis. Arterioscler Thromb Vasc Biol 25(6):1256–1261 15831805 10.1161/01.ATV.0000166517.18801.a7

[CR54] Shankman LS, Gomez D, Cherepanova OA, Salmon M, Alencar GF, Haskins RM, Swiatlowska P, Newman AAC, Greene ES, Straub AC, Isakson B, Randolph GJ, Owens GK (2015) KLF4-dependent phenotypic modulation of smooth muscle cells has a key role in atherosclerotic plaque pathogenesis. Nat Med 21(6):628–637 25985364 10.1038/nm.3866 PMC4552085

[CR55] Sokol RJ, Wales J, Hudson G, Goldstein DJ, James NT (1991) Changes in cellular dry mass during macrophage development. Cells Tissues Organs 142(3):246–248 10.1159/000147197 1796740

[CR56] Swirski FK, Pittet MJ, Kircher MF, Aikawa E, Jaffer FA, Libby P, Weissleder R (2006) Monocyte accumulation in mouse atherogenesis is progressive and proportional to extent of disease. Proc Natl Acad Sci U S A 103(27):10340–10345 16801531 10.1073/pnas.0604260103 PMC1502459

[CR57] Tabas I (2010) Macrophage death and defective inflammation resolution in atherosclerosis. Nat Rev Immunol 10(1):36–46 19960040 10.1038/nri2675 PMC2854623

[CR58] Tang J, Lobatto ME, Hassing L, Staay S, Rijs SM, Calcagno C, Braza MS, Baxter S, Fay F, Sanchez-Gaytan BL, Duivenvoorden R, Sager H, Astudillo YM, Leong W, Ramachandran S, Storm G, Pérez-Medina C, Reiner T, Cormode DP, Strijkers GJ, Stroes ES, Swirski FK, Nahrendorf M, Fisher EA, Fayad ZA, Mulder WJ (2015) Inhibiting macrophage proliferation suppresses atherosclerotic plaque inflammation. Sci Adv 1(3):1400223 10.1126/sciadv.1400223 PMC4539616 26295063

[CR59] Thorp E, Tabas I (2009) Mechanisms and consequences of efferocytosis in advanced atherosclerosis. J Leukoc Biol 86(5):1089–1095 19414539 10.1189/jlb.0209115 PMC2774877

[CR60] Vengrenyuk Y, Nishi H, Long X, Ouimet M, Savji N, Martinez FO, Cassella CP, Moore KJ, Ramsey SA, Miano JM, Fisher EA (2015) Cholesterol loading reprograms the microRNA-143/145-myocardin axis to convert aortic smooth muscle cells to a dysfunctional macrophage-like phenotype. Arterioscler Thromb Vasc Biol 35(3):535–546 25573853 10.1161/ATVBAHA.114.304029 PMC4344402

[CR61] Virmani R, Kolodgie FD, Burke AP, Farb A, Schwartz SM (2000) Lessons from sudden coronary death. Arterioscler Thromb Vasc Biol 20(5):1262–1275 10807742 10.1161/01.atv.20.5.1262

[CR62] Wang Y, Dubland JA, Allahverdian S, Asonye E, Sahin B, Jaw JE, Sin DD, Seidman MA, Leeper NJ, Francis GA (2019) Smooth muscle cells contribute the majority of foam cells in ApoE (Apolipoprotein E)-deficient mouse atherosclerosis. Arterioscler Thromb Vasc Biol 39(5):876–887 30786740 10.1161/ATVBAHA.119.312434 PMC6482082

[CR63] Watson MG, Byrne HM, Macaskill C, Myerscough MR (2018) A two-phase model of early fibrous cap formation in atherosclerosis. J Theor Biol 456:123–136 30098319 10.1016/j.jtbi.2018.08.010

[CR64] Watson MG, Chambers KL, Myerscough MR (2023) A lipid-structured model of atherosclerotic plaque macrophages with lipid-dependent kinetics. Bull Math Biol 85:85 37581687 10.1007/s11538-023-01193-w PMC10427559

[CR65] (WHO) W.H.O. (2021) Cardiovascular Diseases (CVDs). https://www.who.int/news-room/fact-sheets/detail/cardiovascular-diseases-(cvds). Accessed: 2024-09-04

[CR66] Williams JW, Martel C, Potteaux S, Esaulova E, Ingersoll MA, Elvington A, Saunders BT, Huang LH, Habenicht AJ, Zinselmeyer BH, Randolph GJ (2018) Limited macrophage positional dynamics in progressing or regressing murine atherosclerotic plaques brief report. Arterioscler Thromb Vasc Biol 38(8):1702–1710 29903736 10.1161/ATVBAHA.118.311319 PMC6202234

[CR67] Yona S, Kim KW, Wolf Y, Mildner A, Varol D, Breker M, Strauss-Ayali D, Viukov S, Guilliams M, Misharin A, Hume DA, Perlman H, Malissen B, Zelzer E, Jung S (2013) Fate mapping reveals origins and dynamics of monocytes and tissue macrophages under homeostasis. Immunity 38(1):79–91 23273845 10.1016/j.immuni.2012.12.001 PMC3908543

[CR68] Zhou X, Hansson GK (1999) Detection of B cells and proinflammatory cytokines in atherosclerotic plaques of hypercholesterolaemic apolipoprotein E knockout mice. Scand J Immunol 50(1):25–30 10.1046/j.1365-3083.1999.00559.x 10404048

